# Delayed maturation of GABAergic signaling in the *Scn1a* and *Scn1b* mouse models of Dravet Syndrome

**DOI:** 10.1038/s41598-019-42191-0

**Published:** 2019-04-17

**Authors:** Yukun Yuan, Heather A. O’Malley, Melissa A. Smaldino, Alexandra A. Bouza, Jacob M. Hull, Lori L. Isom

**Affiliations:** 1 Department of Pharmacology, University of Michigan, Ann Arbor, MI 48109-5632 USA; 2 Neuroscience Graduate Program, University of Michigan, Ann Arbor, MI 48109-2215 USA; 3 Present Address: Department of Biology, Ball State University, Muncie, IN 47306 USA

**Keywords:** Epilepsy, Neurophysiology

## Abstract

Dravet syndrome (DS) is a catastrophic developmental and epileptic encephalopathy characterized by severe, pharmacoresistant seizures and the highest risk of Sudden Unexpected Death in Epilepsy (SUDEP) of all epilepsy syndromes. Here, we investigated the time course of maturation of neuronal GABAergic signaling in the *Scn1b*^−/−^ and *Scn1a*^+/−^ mouse models of DS. We found that GABAergic signaling remains immature in both DS models, with a depolarized reversal potential for GABA_A_-evoked currents compared to wildtype in the third postnatal week. Treatment of *Scn1b*^−/−^ mice with bumetanide resulted in a delay in SUDEP onset compared to controls in a subset of mice, without prevention of seizure activity or amelioration of failure to thrive. We propose that delayed maturation of GABAergic signaling may contribute to epileptogenesis in *SCN1B*- and *SCN1A*-linked DS. Thus, targeting the polarity of GABAergic signaling in brain may be an effective therapeutic strategy to reduce SUDEP risk in DS.

## Introduction

Dravet syndrome (DS) is a devastating developmental and epileptic encephalopathy (DEE). DS patients develop severe seizures of multiple etiologies in the first year of life^1,2^. In addition to seizures, DS encompasses a range of neurological syndromes including intellectual disability, ataxia, autism spectrum disorders, abnormalities of circadian rhythm, and developmental delay at all milestones^3–5^. Most critically, DS patients have the highest risk of Sudden Unexpected Death in Epilepsy (SUDEP) of all epilepsy syndromes^6,7^. DS mouse models recapitulate many patient phenotypes including severe seizures, developmental delay, ataxia, sleep disorders, and SUDEP^8–13^.

The majority of reported DS cases are linked to *de novo* variants in *SCN1A*, encoding the voltage-gated sodium channel (VGSC) Na_v_1.1 subunit, resulting in haploinsufficiency. A growing list of other gene variants, including in *SCN1B*, encoding the VGSC β1/β1B subunits, has been implicated in DS or DS-like DEEs^14^. Variants in *SCN1B* are the only reported recessive DS linkage; thus, inheritance of mutant *SCN1B* alleles may be subject to genetic counseling and testing. At least seven DS patients with homozygous, recessive mutations in *SCN1B* have been reported to date, with one, *SCN1B-R125C*, shown to be loss-of-function^15–18^. Other *SCN1B* variants of uncertain significance linked to epilepsy and to cardiac disease are reported in ClinVar (https://www.ncbi.nlm.nih.gov/clinvar/). *Scn1b*^−/−^ mice model DS, exhibiting spontaneous seizures beginning at approximately postnatal day (P)10 and death of all animals by ~P21^8^. *Scn1b*^−/−^ mouse brains show aberrant neuronal proliferation, migration, pathfinding, and fasciculation prior to seizure onset, suggesting that these defects may contribute to disease^13^. *Scn1b*^−/−^ mice also have cardiac arrhythmias^19,20^, suggesting possible neuro-cardiac SUDEP mechanisms.

In previous work, we recorded giant depolarizing potentials (GDPs) in *Scn1b*^−/−^ mouse brains at P16^13^. GDPs are synchronized, network-driven synaptic responses that occur during early brain development, mediated in large part by the excitatory action of γ-aminobutyric acid (GABA) on GABA_A_ receptors^21^. Excitatory GABAergic signaling observed in normal mice during the first two postnatal weeks is a consequence of a high intracellular concentration of Cl^−^ ([Cl^−^]_i_) in immature neurons that leads to depolarization in response to opening of GABA_A_ channels. Age-dependent differential expression of the Cl^−^-cation co-transporters (CCCs) Na^+^, K^+^-2Cl^−^ co-transporter-1 (NKCC1) and K^+^-Cl^−^ co-transporter-2 (KCC2) is a primary mechanism of neuronal [Cl^−^]_i_ regulation^21^. NKCC1, which promotes Cl^−^ influx, is expressed in immature neurons and becomes downregulated during brain development^22^. In contrast, KCC2, which extrudes Cl^−^, is expressed at low levels at birth and then upregulated to adult levels by the end of the second postnatal week in rodent brain^23^. The ratio of KCC2/NKCC1 expression thus favors high [Cl^−^]_i_ in immature neurons and low [Cl^−^]_i_ in more mature neurons; as a result, activation of GABA_A_ receptors results in Cl^−^ efflux and membrane depolarization in early brain development and then, as development progresses, Cl^−^ influx and hyperpolarization. Consistent with this developmental shift, GDPs in wildtype (WT) rodent brain disappear after the second postnatal week.

Here, we investigated the maturation time course of neuronal GABAergic signaling in *Scn1b*^−/−^ and *Scn1a*^+/−^ DS mice compared to WT littermates. We show that the reversal potential for GABA_A_-evoked currents (*E*_*GABA*_) is more depolarized compared to WT in the hippocampus and neocortex of *Scn1b*^−/−^ mouse brain at P16, suggesting a developmental delay in the maturation of GABAergic signaling^13^. A similar result, although of lesser magnitude, occurs in *Scn1a*^+/−^ mice. Treatment with the NKCC1 antagonist bumetanide prolongs the lifespan of a subset of *Scn1b*^−/−^ mice, suggesting that therapeutic strategies targeting GABAergic signaling polarity may be useful in reducing SUDEP risk in DS.

## Results

### Maturation of GABAergic signaling is delayed in *Scn1b*^−/−^ brain

**Figure 1:**
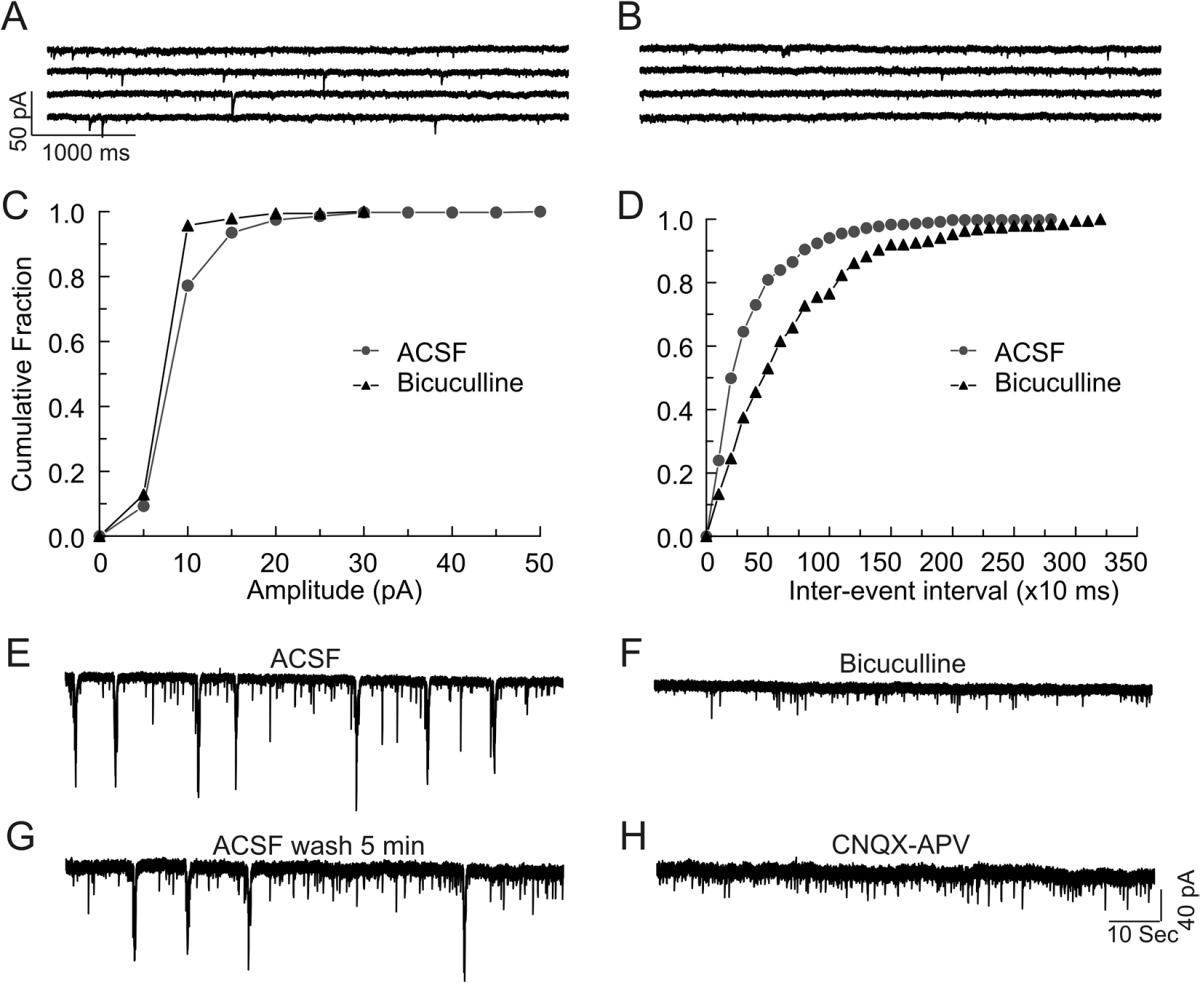
Hippocampal and cortical neurons in brain slices from P4-7 *Scn1b* WT and −/− mice display excitatory GABAergic spontaneous synaptic currents and synchronous recurrent giant inward currents. (**A**–**D**) Spontaneous postsynaptic currents in cortical layer II/III and hippocampal CA1 or CA3 pyramidal cells from P4-7 *Scn1b* WT or −/− mice were recorded at a holding potential of −70 mV using the whole-cell patch-clamp recording technique, a low Cl^−^-containing, K-gluconate-based internal solution, and ACSF external solution. (**A**,**B**) Representative recording of spontaneous postsynaptic currents in a hippocampal CA3 pyramidal cell from a P7 WT mouse in the absence (**A**) and presence (**B**) of 10 µM bicuculline. (**C**) Bicuculline blocked large amplitude inward currents and shifted the curve of cumulative amplitude distribution to the left. (**D**) Bicuculline reduced the frequency of synaptic currents and shifted the curve of cumulative inter-event interval distribution to the right. Similar results were seen in hippocampal CA1 and cortical layer II/III neurons in slices from both *Scn1b* WT and −/− mice. Each value is representative of 5–6 individual mice. (**E**–**H**) Spontaneous GDP-related giant inward currents were recorded from *Scn1b* WT or −/− hippocampal CA3 pyramidal cells at a holding potential of −70 mV using a NMDG-Cl-based pipette solution. (**E**) Representative currents from P6 WT brain. (**F**) Giant currents are sensitive to block by bicuculline. (**G**) Block by bicuculline is reversible. (**H**) Giant inward currents are sensitive to block by glutamate receptor antagonists CNQX and APV. Each trace is representative of 7 CA3 neurons from 4 individual WT mice.

**Figure 2:**
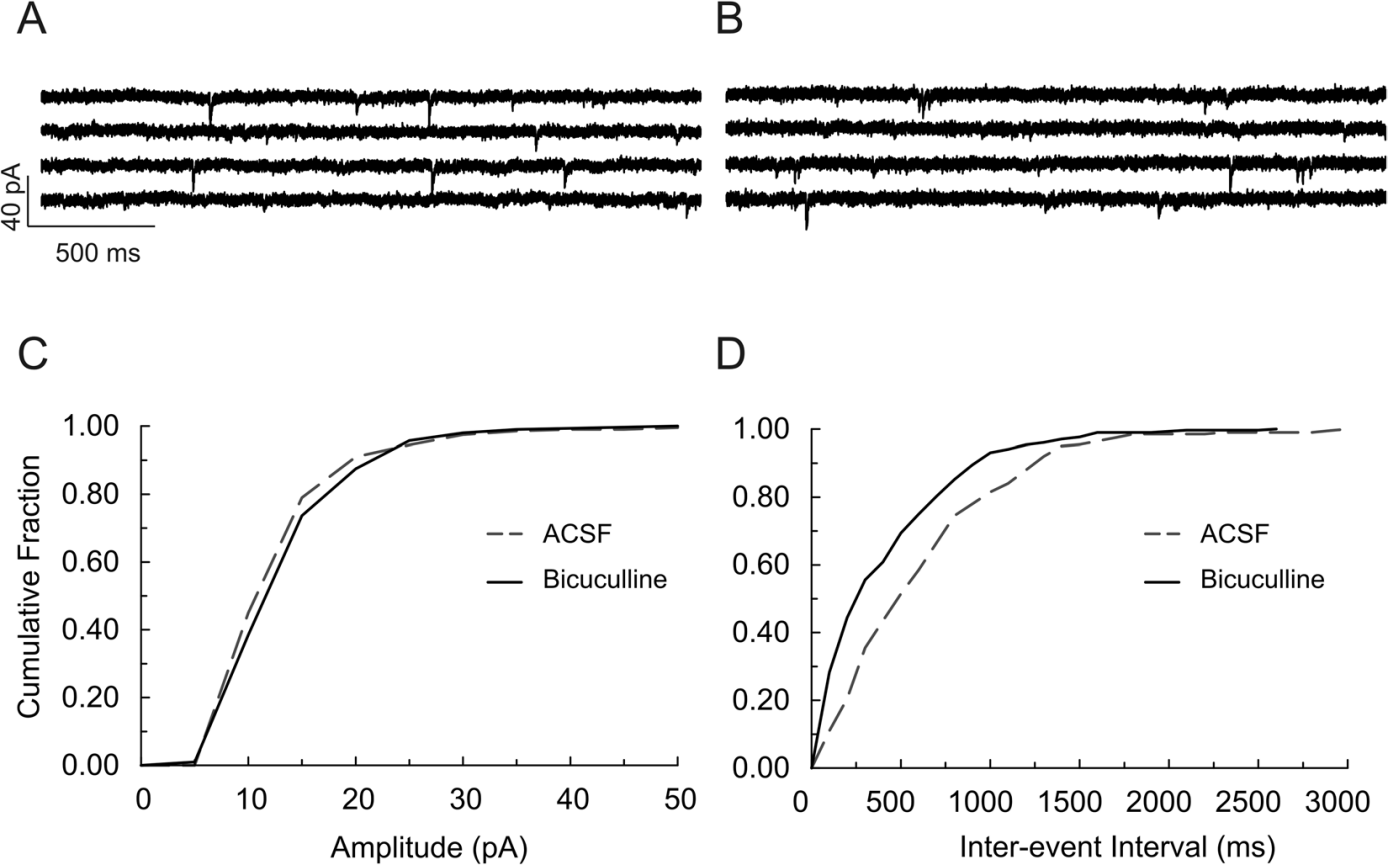
GABAergic currents in cortical and hippocampal neurons of *Scn1b* WT mice are outward/hyperpolarizing after the second postnatal week. Whole cell bicuculline-sensitive inward currents were recorded from cortical layer II/III and hippocampal CA1 or CA3 neurons of P13-19 *Scn1b* WT mice using a low Cl^–^ containing, K-gluconate-based pipette solution at a holding potential of −70 mV. (**A**,**B**) Representative recordings of spontaneous synaptic inward currents in a cortical layer II/III pyramidal cell of a P17 *Scn1b* WT mouse in the absence (**A**) and presence (**B**) of 10 µM bicuculline. Bicuculline had no significant effect on the amplitudes of synaptic events (**C**), but significantly increased the frequency of synaptic responses (**D**). Each value is representative of 5–10 individual mice.

**Figure 3:**
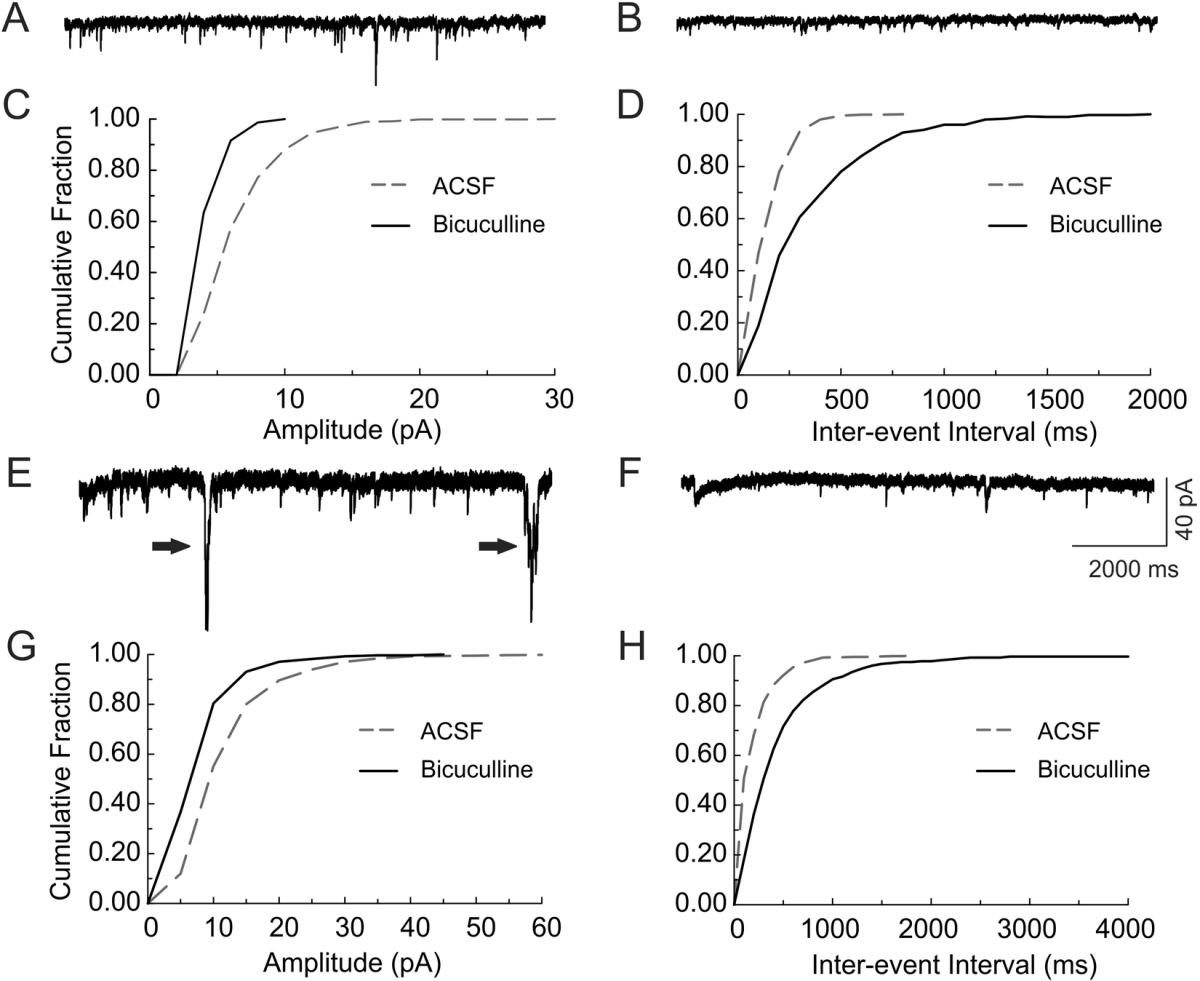
GABAergic currents in cortical and hippocampal neurons of *Scn1b*^−/−^ mice remain inward/depolarizing after the second postnatal week. Whole cell recordings of bicuculline-sensitive inward currents were made from P13-19 *Scn1b*^−/−^ brain slices under similar conditions as described in Fig. 2. (**A**–**D**) Representative recordings of spontaneous synaptic inward currents in a cortical layer II/III pyramidal cell of a P17 *Scn1b*^−/−^ mouse in the absence (**A**) and presence (**B**) of 10 µM bicuculline. Bicuculline blocked all large amplitude, inward synaptic events (**C**) and significantly decreased the frequency of synaptic responses (**D**). Each value is representative of 10 individual mice. (**E**–**H**) Representative recordings of spontaneous synaptic inward currents in a CA3 pyramidal cell of a P18 *Scn1b*^−/−^ mouse in the absence (**E**) and presence (**F**) of 10 µM bicuculline. Bicuculline blocked all large amplitude, inward synaptic events (**G**) and significantly decreased the frequency of synaptic responses. (**H**) Arrows in E indicate large amplitude inward currents corresponding to GDPs. Each value is representative of 7–10 individual mice.

To determine the time course of maturation of GABAergic signaling polarity in *Scn1b* WT and −/− brain, we examined spontaneous postsynaptic responses in neocortical layer II/III and hippocampal CA1 or CA3 pyramidal cells in acute brain slices prepared from P4-7 mice (Fig. 1, panels A–D). As expected for early excitatory actions of GABA^21^, a large number of spontaneous postsynaptic events recorded in brain slices from both genotypes over this time range were bicuculline-sensitive inward currents in the presence of a low Cl^–^containing, K-gluconate-based internal solution (Fig. 1A), reflecting excitatory GABAergic responses. Bicuculline blocked all large amplitude inward currents and reduced the frequency of spontaneous synaptic events (Fig. 1B), shifting the cumulative amplitude distribution curve to the left (Fig. 1C) and shifting the cumulative inter-event interval (frequency) curve to the right (Fig. 1D). These inward currents were thus mediated by GABA_A_ receptors. Consistent with our previous work^13^, hippocampal CA3 pyramidal neurons in P4-7 WT brain slices showed synchronized, recurrent, slow giant inward currents, corresponding to GDPs, when recorded using a NMDG-based internal solution (Fig. 1, panels E and G). The rate of occurrence of giant inward currents was 0.09 ± 0.01 Hz (n = 14), similar to previous findings^24^. Giant inward currents were sensitive to block by bicuculline (Fig. 1F) or the glutamate receptor antagonists CNQX and APV (Fig. 1H), consistent with GDPs being GABA_A_ receptor-mediated responses and a result of network-driven, synergistic action of both GABA and glutamate^21^. As shown previously^24^, no detectable bicuculline-sensitive spontaneous inward current events were observed when recorded under similar conditions in WT brain slices at P13–19 (Fig. 2, panels A and B), although infrequent bicuculline-sensitive currents could be recorded as upward events in both cortical and hippocampal neurons (Fig. S1A). Fig. S1 shows representative differences in bicuculline-sensitive GABAergic currents between P16-17 *Scn1b* WT and −/− CA1 neurons. WT GABAergic currents were outward events because the holding potential for recording spontaneous synaptic currents was more depolarized than the predicted *E*_*GABA*_ (−83 mV) (Fig. S1A). In contrast, bicuculline-sensitive currents in *Scn1b*^−/−^ neurons were inward events with larger amplitudes, suggesting that *E*_*GABA*_ was more depolarized than the holding potential for recording synaptic responses (Fig. S1B). Application of bicuculline to WT P13-19 slices had no significant effects on the amplitudes of spontaneous synaptic inward events (Figs 2C and S1A) but blocked all outward synaptic events and significantly increased the frequency of spontaneous inward synaptic currents (Fig. 2D), suggesting increased glutamate release due to GABA_A_ receptor blockade. These data show that GABAergic synaptic responses in WT mice were mature and inhibitory by ~P13. In contrast, ~70% of recordings from P13–19 *Scn1b*^−/−^ cortical layer II/III (Fig. 3, panels A–D) or hippocampal CA1 (Fig. S1B) or CA3 (Fig. 3E-H) pyramidal neurons exhibited large amplitude, spontaneous inward synaptic currents. Application of bicuculline reversibly blocked these inward synaptic currents (Fig. 3, panels C and G, and Fig. S1B) and significantly reduced the frequency of synaptic events (Fig. 3, panels D and H, and Fig. S1B), suggesting that these inward currents were mediated by GABA_A_ receptors. Notably, large amplitude inward currents corresponding to GDPs remained observable and bicuculline-sensitive in CA3 pyramidal cells of *Scn1b*^−/−^ mice over this time range (Fig. 3E, arrows). Taken together, these data show that GABAergic function remains immature, inward, and depolarizing from P13-19 in *Scn1b*^−/−^ brain while GABAergic function over this same time range in WT littermates is outward and hyperpolarizing.

### *Scn1b*^−/−^ neurons have a depolarized reversal potential for GABA

**Figure 4:**
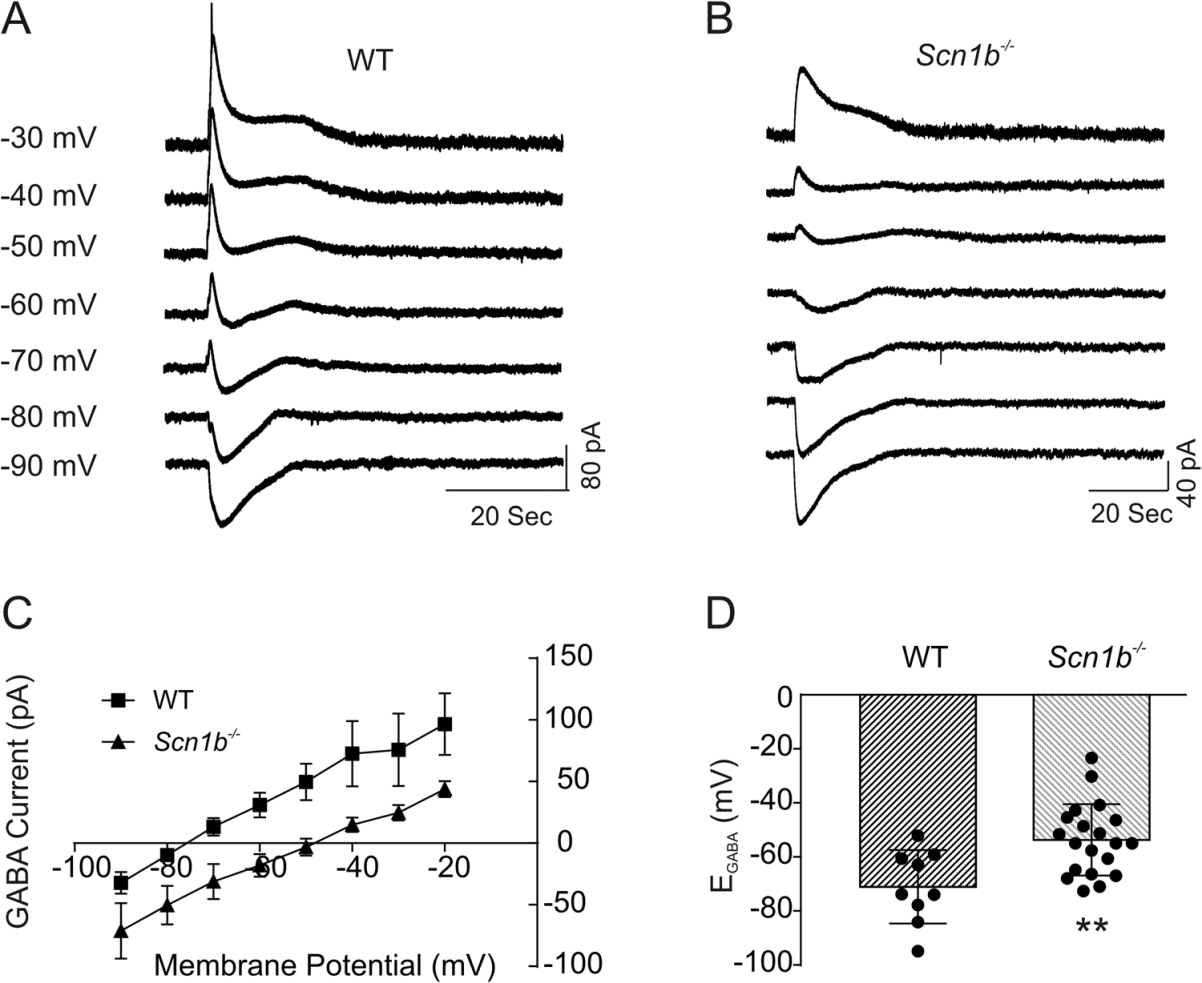
GABA reversal potentials in *Scn1b*^−/−^ neurons are significantly depolarized compared to WT. GABA-evoked currents at different holding potentials were recorded using the gramicidin perforated patch clamp method from cortical layer II/III and hippocampal CA1 and CA3 neurons in slices from P13-19 *Scn1b* WT or −/− mice. (**A**,**B**) Representative GABA currents evoked in a layer II/III pyramidal cell in a cortical slice of a P17 *Scn1b* WT (**A**) or −/− (**B**) mouse at holding potentials varying from −90 mV to −30 mV. (**C**) Comparison of the current-voltage relationships (I-V curves) obtained from *Scn1b* WT and −/− mice. Values are pooled from cortical and hippocampal neurons. (**D**) Comparison of reversal potentials for GABA-mediated currents in *Scn1b* WT and −/− neurons. Each value is representative of 6–7 individual mice.

Our observation of bicuculline-sensitive inward currents in P13-19 *Scn1b*^−/−^ brain slices suggested a developmental delay in the maturation of neuronal Cl^−^ gradients and GABAergic signaling. To test this hypothesis, we measured *E*_*GABA*_ in P13-19 *Scn1b* WT and −/− brain slices using the gramicidin perforated patch recording technique (Fig. 4, panels A and B). We found no significant differences in *E*_*GABA*_ between cortical and hippocampal pyramidal cells within each genotype; thus, the data presented were pooled from cells in these brain regions. The mean *E*_*GABA*_ (−53.3 ± 3.0 mV, n = 20) of neurons in slices from P13-19 *Scn1b*^−/−^ mice was significantly more depolarized than in slices from age-matched WT mice (−72.4 ± 4.3 mV, n = 10, p < 0.01, Fig. 4, panels C and D). Because the majority of *Scn1b*^−/−^ animals die at ~P17-19, it was not possible to measure *E*_*GABA*_ at later time points with statistical significance. However, dividing the data into two age groups, P13-17 and P18-19, shows that the mean *E*_*GABA*_ values are −66.7 ± 5.6 mV (n = 5) for WT and −51.1 ± 3.6 mV (n = 12) for −/− neurons at P13-17 and −77.8 ± 5.9 mV (n = 5) for WT and −57.9 ± 6.7 mV (n = 6) for −/− neurons at P18-19. Approximately 30% of recordings of sEPSCs in P18-19 cortical, CA1, or CA3 pyramidal −/− neurons did not show bicuculline-sensitive inward currents (data not shown). Thus, the value of *E*_*GABA*_ for *Scn1b*^−/−^ neurons may become more hyperpolarized toward the time of SUDEP. Taken together, these results suggest that the developmental switch of GABAergic function from excitation to inhibition is prevented or delayed in *Scn1b*^−/−^ brain.

### KCC2 and NKCC1 are expressed at normal levels in *Scn1b*^−/−^ and *Scn1a*^+/−^ brains

**Figure 5:**
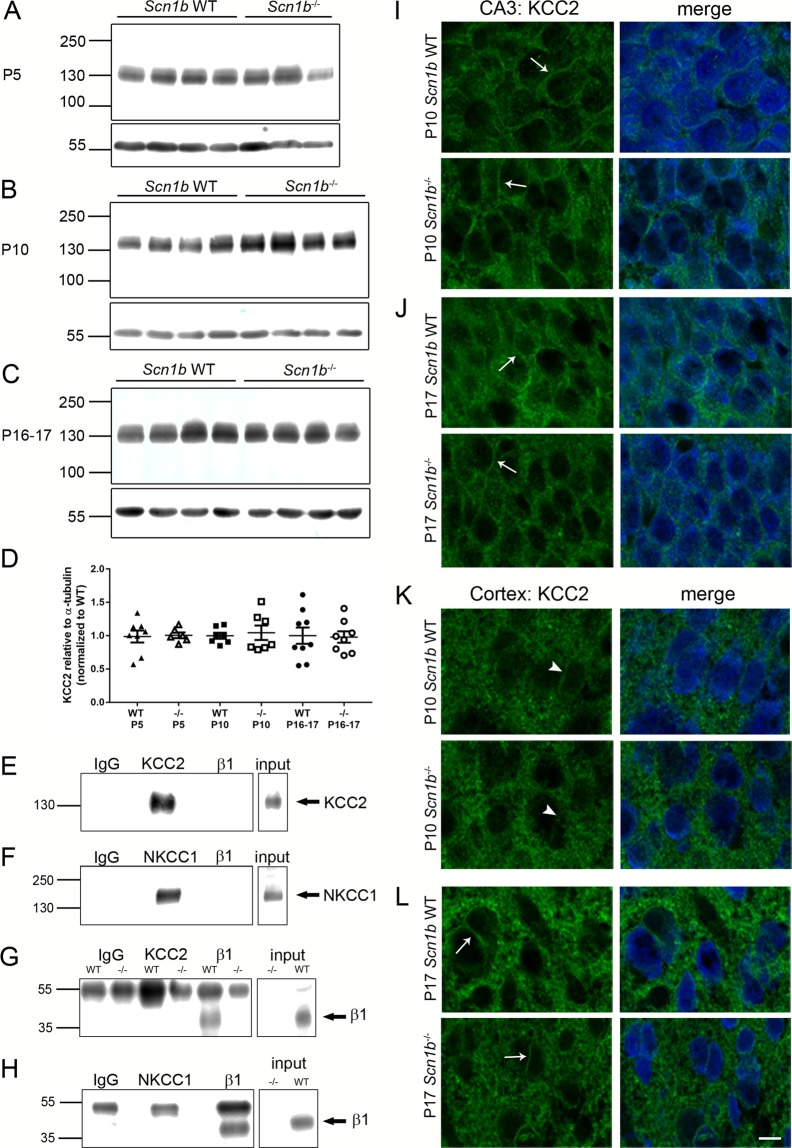
KCC2 expression levels are similar in *Scn1b* WT and −/− brain, KCC2 subcellular localization is similar in *Scn1b* WT and −/− neurons, and neither KCC2 nor NKCC1 associate with β1 subunits in WT brain. (**A**–**C**) Western blots of KCC2 protein levels with α-tubulin loading controls in *Scn1b* WT and −/− brain at 3 developmental stages. (**A**) KCC2 expression at P5. (**B**) KCC2 expression at P10. (**C**) KCC2 expression at P16-17. (**D**) Quantification of Western blot results at all 3 postnatal ages relative to α-tubulin loading controls and normalized to WT. (**E**–**H**) Co-immunoprecipitation of β1 and either KCC2 or NKCC1 in WT brain demonstrates no association between β1 and either co-transporter protein. (**E**,**G**) Anti-KCC2 immunoprecipitates KCC2 polypeptides (**E**) but does not co-immunoprecipitate β1 (**G**). (**F**,**H**) Anti-NKCC1 immunoprecipitates NKCC1 polypeptides (**F**) but does not co-immunoprecipitate β1 (**H**). Anti-β1 immunoprecipitates β1 polypeptides (**G**,**H**). (**I–L**) Immunofluorescent images showing localization of KCC2 in hippocampal CA3 or cortical layer II/III neurons at 2 developmental stages. (**I**,**J**) KCC2 localizes near the plasma membrane (arrows) in the majority of *Scn1b* WT and −/− CA3 neurons at P10 (**I**) and P17 (**J**). (**K**,**L**) KCC2 does not display significant plasma membrane localization (arrowheads) in cortical layer II/III neurons from both genotypes at P10 (**K**) but does display localization near to the plasma membrane (arrows) in a subset of neurons from both genotypes at P17 (**L**). Green: KCC2, blue: NeuN. Scale bar = 20 µm.

An important mechanism controlling [Cl^−^]_i_ in brain neurons is the relative protein expression levels of KCC2 and NKCC1^22,23^. We performed Western blot analyses of total brain membranes (intracellular and extracellular) from *Scn1b* WT and −/− mice to determine whether the protein expression levels of these CCCs were altered as a result of *Scn1b* deletion (Fig. 5, panels A–C). Three ages were compared for each genotype: P5 (Fig. 5A, prior to seizure onset), P10 (Fig. 5B, just prior to or at seizure onset), and P16-P17 (Fig. 5C, post-seizure onset). Figure 5D shows the quantification of immunoreactive band densities relative to α-tubulin loading controls and normalized to WT. We found no differences in total KCC2 protein expression between genotypes at any postnatal age (P5: p = 0.7546, WT n = 8, −/− n = 6; P10: p = 0.8048, n = 7 for both genotypes; P16-17: p > 0.9999, WT n = 9, −/− n = 8; all samples run in duplicate). Although we performed similar Western blot experiments using two different commercially available antibodies against NKCC1, the immunoreactive bands either did not display reliable signals across samples of the same genotype or did not result in bands suitable for quantification (data not shown). Thus, relative levels of NKCC1 expression at various postnatal ages and the ratio of NKCC1: KCC2 expression between genotypes could not be evaluated. Comparison of relative mRNA transcript levels in *Scn1b* WT and −/− brain at P17 using real-time quantitative RT-PCR showed no differences between genotypes for *Slc12a5* (KCC2) (Fig. S2A, p = 0.2086, n = 7 per genotype) or *Slc12a2* (NKCC1) (Fig. S2B, p = 0.6200, n = 7 per genotype). Because most reported cases of DS are linked to *SCN1A* haploinsufficiency, we also evaluated transcript levels of *Scn1a* in *Scn1b* −/− and WT brains but found no difference between genotypes (Fig. S2C, p = 0.2086, n = 7 per genotype).

Taking an unbiased approach, we performed RNAseq experiments in cortical layer VI of P10 *Scn1b* WT and −/− mouse brains (Fig. S2, panels D and E). 21 differentially expressed genes were identified (log2 fold change ≥1.5 and false discovery rate adjusted p-value ≤ 0.05). 19 genes were downregulated in the −/− samples compared to WT, while two were upregulated (Fig. S2D). However, transcripts encoding NKCC1 and KCC2, *Slc12a2* and *Slc12a5* respectively, were not differentially expressed between genotypes, in agreement with Fig. S2. Similarly, transcripts encoding kinases known to regulate KCC2 abundance at the plasma membrane, *WNK1* and *STK39*, were not differentially expressed between genotypes (Fig. S2E).

We performed co-immunoprecipitation experiments using solubilized *Scn1b* WT total brain membranes to determine whether VGSC β1 subunits normally associate with KCC2 or NKCC1 in brain in any subcellular compartment (Fig. 5, panels E–H). Immunoprecipitation with anti-β1 followed by immunoblotting with anti-KCC2 (Fig. 5E) or immunoprecipitation with anti-KCC2 followed by immunoblotting with anti-β1 (Fig. 5G) showed no evidence for association of these two proteins in brain. Similarly, immunoprecipitation with anti-β1 followed by immunoblotting with anti-NKCC1 (Fig. 5F) or immunoprecipitation with anti-NKCC1 followed by immunoblotting with anti-β1 (Fig. 5H) showed no evidence for association of these two proteins in brain. As positive controls, anti-KCC2 (Fig. 5E), anti-NKCC1 (Fig. 5F), and anti-β1 (Fig. 5G,H) all showed robust immunoprecipitation of their own antigens.

We compared KCC2 and NKCC1 mRNA and protein expression levels in *Scn1a* WT and +*/−* brains (Fig. S3). Similar to results in *Scn1b*^−/−^ brain (Fig. 5), there was no difference in KCC2 protein expression between genotypes at P16 (Fig. S3A, p = 0.9497, WT: n = 6, +*/−*: n = 8). We evaluated transcript levels for *Slc12a5* (KCC2) with no difference between genotypes at P16 (Fig. S3D, p = 0.9372, n = 6 per genotype) or P22 (Fig. S3G, p = 0.6991, n = 6 per genotype). Transcript levels for *Slc12a2* (NKCC1) showed no difference between genotypes at P16 (Fig. S3E, p = 0.3095, n = 6 per genotype) or P22 (Fig. S3H, p = 0.8182, n = 6 per genotype).

Protein expression levels of β1 were not different between *Scn1a* WT and +*/−* mice at P16-17 (Fig. S3B, p = 0.1487, WT: n = 16, +*/−*: n = 24) or P21-24 (Fig. S3C, p = 0.8421, WT: n = 9, +*/−*: n = 10). A larger sample size was used at P16-17 to determine whether two different populations of β1 protein expression could be detected in *Scn1a*^+/−^ mice, since not all mice in this model develop the DS phenotype. However, no significant intra-group differences were observed. Relative transcript levels of *Scn1b* were not different between genotypes at P16 (Fig. S3F, p = 0.6991, n = 6 per genotype) or P22 (Fig. S3I, p = 0.1797, n = 6 per genotype).

### No changes in KCC2 subcellular localization between *Scn1b*^−/−^ and WT brain

We performed immunohistochemistry experiments with brain cryosections to determine whether there were differences in the subcellular localization of KCC2 in hippocampal CA3 or cortical layer II/III neurons between genotypes (Fig. 5, panels I–L). Similar to previously published results^25^, at both P10 (Fig. 5I, arrow) and P17 (Fig. 5J, arrow) there was robust localization of KCC2 immunofluorescence near the neuronal plasma membrane in hippocampal CA3 neurons (green: KCC2; blue: NeuN in merged images). While there was minimal localization of KCC2 to the plasma membrane of cortical layer II/III neurons at P10 (Fig. 5K, arrowheads; green: KCC2; blue: NeuN in merged images), by P17 a subset of neurons displayed localization of KCC2 that was in the region of the plasma membrane (Fig. 5L, arrows; green: KCC2; blue: NeuN in merged images) and there was an increase in fluorescence intensity in the neuropil. While previous work has demonstrated cell surface localization of KCC2 using biotinylation techniques^26^, the present results cannot differentiate between cell surface vs. sub-plasma membrane localization. Regardless, there were no observable differences between genotypes at either age or brain region, suggesting that differences in GABAergic signaling in *Scn1b*^−/−^ brain are not caused by deficiencies in subcellular localization of KCC2.

### Bumetanide administration delays SUDEP, but does not prevent seizures, in a subset of *Scn1b*^−/−^ mice

**Figure 6:**
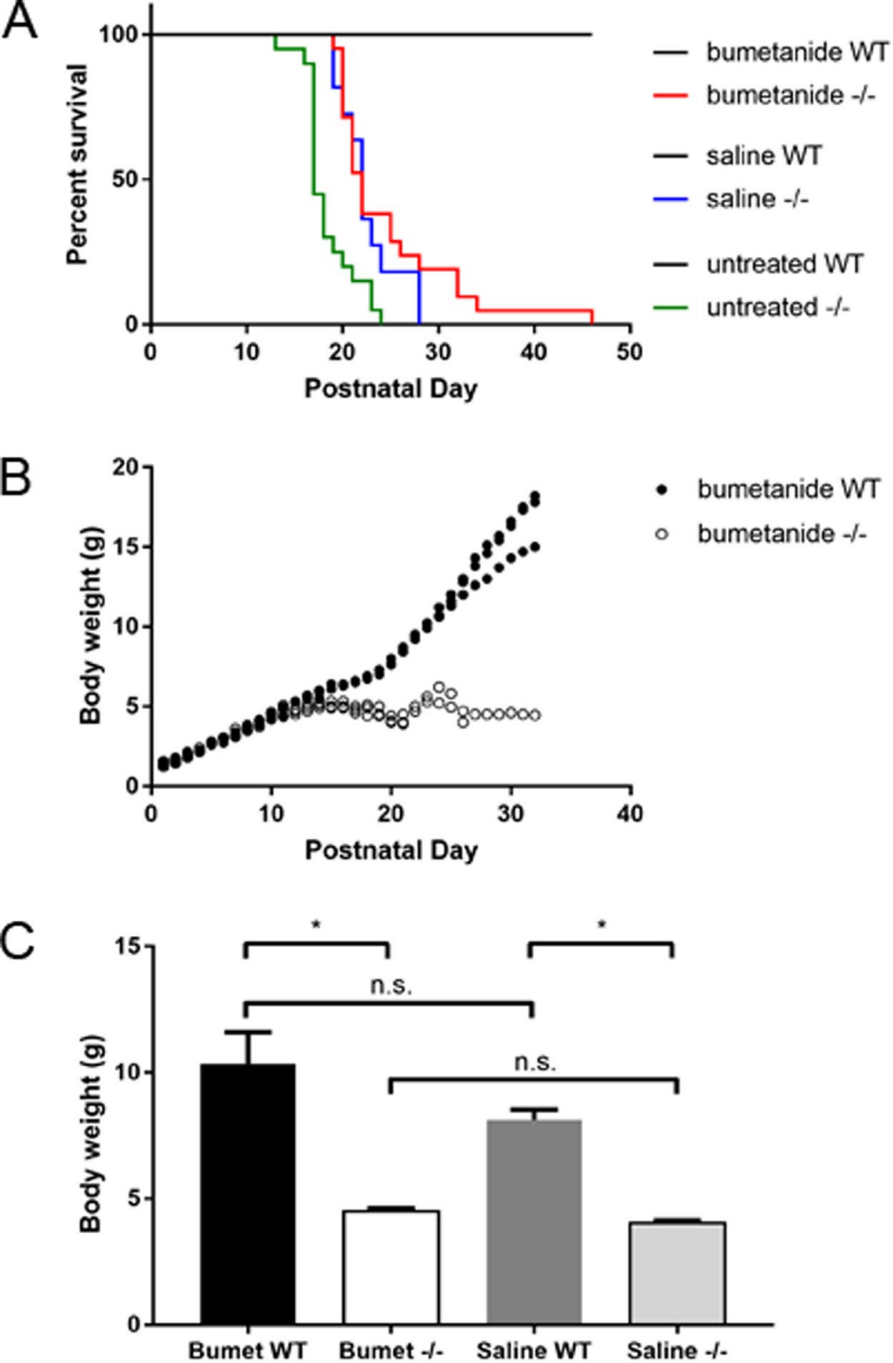
Administration of the NKCC1 antagonist bumetanide delays SUDEP in a subset of *Scn1b*^−/−^ mice. (**A**) Kaplan-Meier survival curve showing survival of bumetanide-treated *Scn1b* WT (black) and −/− (red), saline-treated WT (black) and −/− (blue), and untreated WT (black) and −/− (green) mice. (**B**) Representative graph of body weights of one litter of bumetanide-treated *Scn1b* WT and −/− mice at each postnatal day. (**C**) Comparison of body weight at endpoint (−/−: day of death, WT: day of death of last −/− in that litter) between bumetanide-treated WT and −/− and saline-treated WT and −/− mice. *p < 0.05. One of 19 bumetanide-treated *Scn1b*^+/−^ mice died for unknown reasons during the treatment period.

Because we observed immature GABAergic signaling in *Scn1b*^−/−^ neurons, we hypothesized that treatment with the NKCC1 inhibitor bumetanide might affect disease progression in this mouse model. We treated litters of *Scn1b* mice, beginning at P0-1, twice daily with subcutaneous injections of 0.2 mg/kg bumetanide, following a published protocol^27^, despite evidence in the literature that bumetanide accumulation in mammalian brain is limited^28^. We reported previously that 100% of *Scn1b*^−/−^ mice undergo SUDEP by ~P21^8^. Following treatment with bumetanide, survival of a subset of *Scn1b*^−/−^ mice was significantly prolonged (Fig. 6A, red), with 8 of 21 −/− mice surviving until P25 and as long as P46 (p < 0.0001 vs. saline or untreated. Bumetanide-treated: WT n = 17, −/− n = 21. Total number of litters injected: 8). *Scn1b*^−/−^ mice injected with saline displayed a small prolongation in survival (Fig. 6A, blue) but not to the extent of bumetanide-treated animals (p < 0.0001 vs. bumetanide or untreated. Saline-treated: WT n = 6, −/− n = 11. Total number of litters injected: 5). Untreated *Scn1b*^−/−^ mice died as early as P13, with the majority of animals dying between P17−18 (Fig. 6A, green), consistent with our previous work^8^ (Untreated: WT n = 10, −/−  n=20).

### Bumetanide treatment of *Scn1b*^−/−^ mice does not affect failure to thrive

DS patients show developmental delays, including small stature and failure to thrive^3–5^. *Scn1b*^−/−^ mice similarly fail to thrive, showing small stature and low body weight compared to WT littermates^8^. Although bumetanide administration prolonged the lifespan of a subset of *Scn1b*^−/−^ mice, it did not affect weight gain. *Scn1b*^−/−^ mouse body weights plateaued after the second postnatal week and rarely increased past 5.5 g (Fig. 6B). In contrast, WT littermates showed increased body weight with age (Fig. 6B). Bumetanide- and saline-treated *Scn1b*^−/−^ mice both displayed endpoint body weights that were significantly lower than WT littermates (bumetanide: p = 0.0001, WT vs −/−; saline: p = 0.0001, WT vs −/−), while there were no differences within the WT or −/− groups due to treatment type (bumetanide WT vs. saline WT: p = 0.3770; bumetanide −/− vs. saline −/−: p = 0.9706) (Fig. 6C). Bumetanide treatment did not prevent seizures in *Scn1b*^−/−^ animals. Although mice were too small for electroencephalographic monitoring prior to SUDEP, all treated *Scn1b*^−/−^ mice were observed to experience at least one behavioral seizure of comparable timing and seizure severity relative to saline-treated or untreated *Scn1b*^−/−^ mice. *Scn1b*^+/−^ mice, which do not seize and live normal life spans, had body weights comparable to WT littermates in the bumetanide- and saline-treated groups (bumetanide n = 19, saline n = 11, data not shown).

### Growth Parameters in *SCN1B*-DS patients

The failure to thrive phenotype of *Scn1b*-DS animals models human *SCN1B*-linked DS. Table S1 summarizes the growth parameters (percentile ranking of height, weight, and head circumference) of three previously reported pediatric *SCN1B*-DS patients (two with the homozygous variant c.449-2A > G, thought to be a splice site mutation, and one with the homozygous variant c.355T > G resulting in p.Y119D)^17^. Similar to our mouse data, growth parameters were significantly below normal.

### *Scn1a*^+/−^ neurons have depolarized values of *E*_*GABA*_

**Figure 7:**
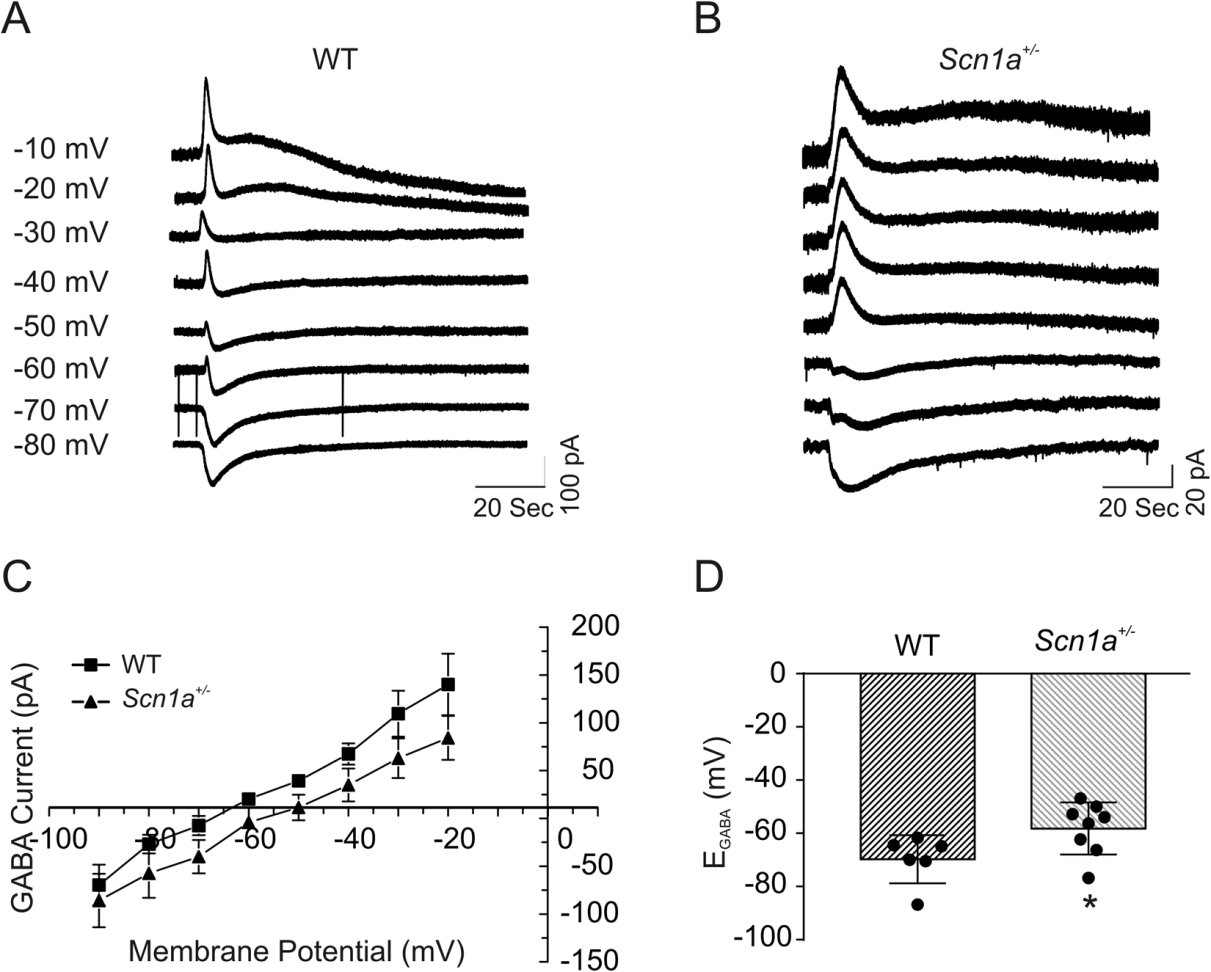
GABA reversal potentials in *Scn1a*^+/−^ neurons are significantly depolarized compared to WT at P13-21. GABA-evoked currents at different holding potentials were recorded using the gramicidin perforated patch clamp method from cortical layer II/III and hippocampal CA1 and CA3 neurons in slices from P13-21 *Scn1a* WT or +*/−* mice. (**A**,**B**) Representative GABA currents evoked in a layer II/III pyramidal cell in a cortical slice from a P17 *Scn1a* WT (**A**) or +/− (**B**) mouse at holding potentials varying from −80 mV to −10 mV. (**C**) Comparison of the current-voltage relationships (I–V curves) obtained from *Scn1a* WT and +*/−* mice. Values are pooled from cortical and hippocampal neurons. (**D**) Comparison of reversal potentials for GABA-mediated currents in *Scn1a* WT vs. +*/−* neurons. Each value is representative of 6–7 individual mice.

To test whether a similar delay in maturation of neuronal GABAergic signaling occurs in a different model of DS, *Scn1a*^+/−^ mice on the (C57BL/6J × 129S6/SvEvTac)F1 strain^29^, we examined *E*_*GABA*_ in P13-21 *Scn1a* WT and +*/−* cortical and hippocampal pyramidal neurons (Fig. 7, panels A and B) using the same procedures as for *Scn1b* neurons in Fig. 4. Similar to our results for *Scn1b*-DS mice, there were no significant differences in *E*_*GABA*_ between neocortical and hippocampal pyramidal neurons within each genotype, thus, the data were pooled from cells in these brain regions. Similar to *Scn1b*^−/−^ mice, we found the mean *E*_*GABA*_ of *Scn1a*^+/−^ neurons to be significantly more depolarized than WT. The mean *E*_*GABA*_ (−58.2 ± 3.5 mV, n = 8) of neurons in slices from P13-21 *Scn1a*^+/−^ mice was significantly more depolarized than in slices from age-matched WT mice (−69.7 ± 3.7 mV, n = 6, p < 0.05, Fig. 7, panels C and D). This time point is just prior to seizure onset in this DS model, typically at ~P18 with SUDEP occurring in ~40–50% of *Scn1a*^+/−^ mice by P22-24^29^, and thus the observation of depolarized *E*_*GABA*_ relative to WT is not compensatory to seizure activity. The mean *E*_*GABA*_ in *Scn1a*^+/−^ neurons was less depolarized than that of *Scn1b*^−/−^ neurons over a similar time range (compare Fig. 7D vs. 4D), suggesting that these two DS models are similar but not identical and that the phenotype in *Scn1b*^−/−^ brain is more severe than in *Scn1a*^+/−^ brain.

### Acute bumetanide application *in vitro* suppresses GABA-evoked excitation

**Figure 8:**
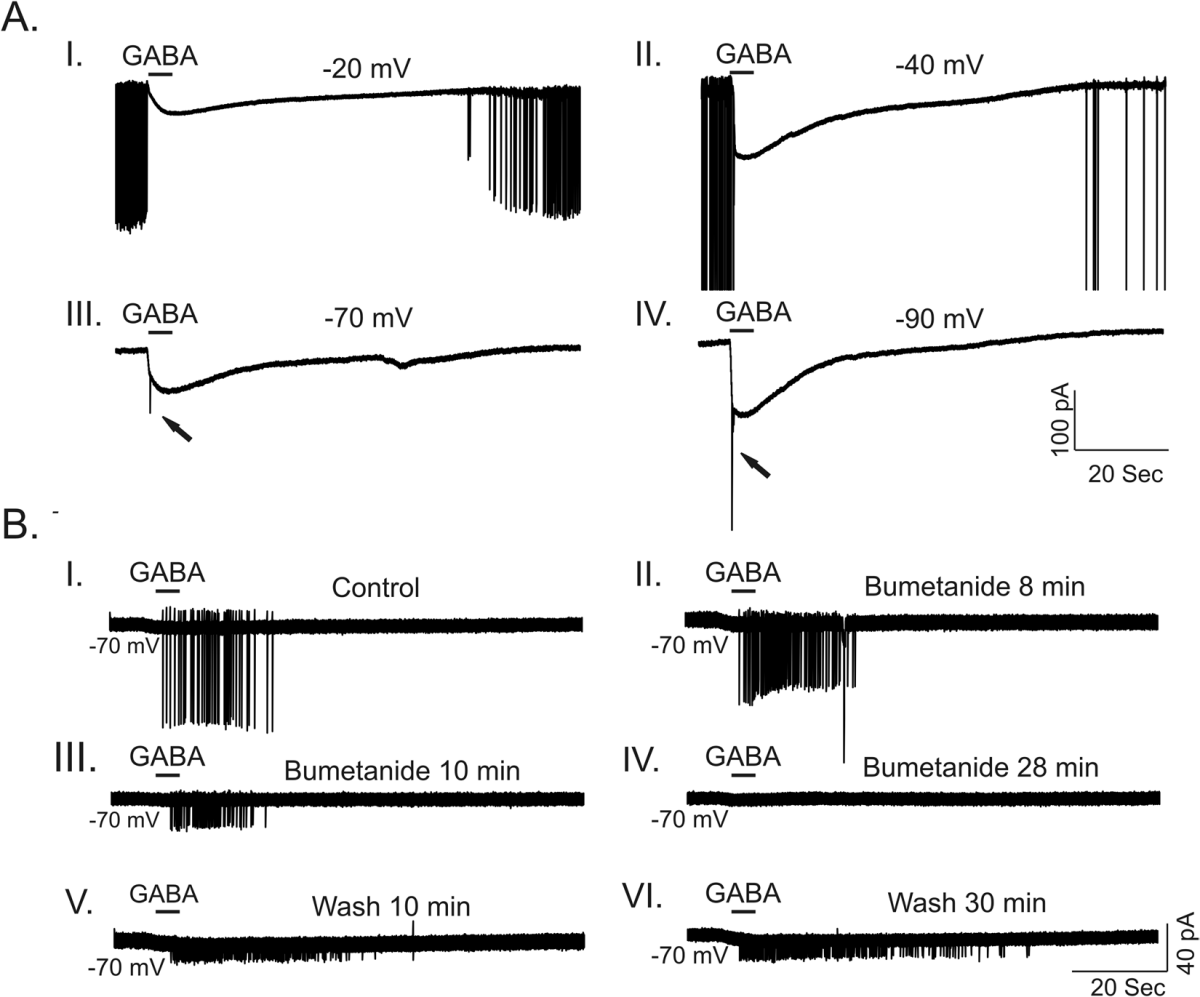
Bumetanide suppresses GABA-evoked firing in P14-18 *Scn1b*^−/−^ neurons. (**A**) Representative traces showing GABA-evoked responses at different membrane holding potentials after establishing gramicidin perforated patch recording. At membrane potentials more depolarized than −60 mV (−20 and −40 mV shown here), *Scn1b*^−/−^ neurons fired spontaneously. Puff application of GABA suppressed spontaneous firing (**A-I** and **A-II**). In contrast, at membrane potentials more hyperpolarized than −60 mV, application of GABA induced AP firing (**A-III** and **A-IV**). (**B**) Representative example of the time course of the effect of 20 µM bumetanide on GABA-induced firing in a P16 *Scn1b*^−/−^ CA1 pyramidal cell. At a holding potential of −70 mV, puff application of GABA induced repetitive firing in a subset of neurons. Bath application of 20 µM bumetanide resulted in the time dependent inhibition of GABA-evoked firing (**B-I** to **B-IV**). 30 min wash with ACSF partially reversed the GABA-evoked firing (**B-V** and **B-VI**). Each trace is representative of results from 4 individual mice.

Both *Scn1b*^−/−^ and *Scn1a*^+/−^ neurons display depolarized *E*_*GABA*_. Thus, we asked whether activation of GABA_A_ receptors results in inhibition or excitation in these DS models. GABA-induced depolarization may lead to shunting inhibition if the value of *E*_*GABA*_ lies between the resting membrane potential and the threshold potential for AP initiation^30^. Using the gramicidin perforated patch technique, we found that the mean resting membrane potentials for *Scn1b*^−/−^ and *Scn1a*^+/−^ neurons were −76.6 ± 2.0 (n = 20) and −70.4 ± 3.4 (n = 8), respectively. Recordings were performed in the presence of TTX to prevent spontaneous AP firing from interfering with our ability to reliably measure GABA-evoked currents at more depolarized membrane holding potentials. Thus, the thresholds for AP generation were not measured. Using conventional whole cell current-clamp recordings from age-matched *Scn1b*^−/−^ neurons, we found that the mean threshold for the initiation of APs was −54.4 ± 3.1 mV (n = 10) and the average resting membrane potential was −64.8 ± 1.9 (n = 10). The value of *E*_*GABA*_ for *Scn1b*^−/−^ neurons was more depolarized than the threshold for AP generation. Application of GABA suppressed spontaneous AP firing at membrane potentials more positive than −60 mV and evoked spikes at membrane potentials more negative than −60 mV (Fig. 8A). GABA application evoked repetitive firing in a subset of *Scn1b*^−/−^ neurons (n = 4). In these cells, acute application of 10–20 μM bumetanide induced a partially reversible inhibition of firing (Fig. 8B). For *Scn1a*^+/−^ neurons, application of GABA resulted in effects similar to those recorded in *Scn1b*^−/−^ neurons (Fig. S4). *Scn1a*^+/−^ neurons often fired spontaneously at more negative membrane potentials (−60 mV to −70 mV) (Fig. S2B) compared to *Scn1b*^−/−^ neurons, suggesting that the threshold for AP generation in *Scn1a*^+/−^ neurons is more hyperpolarized than that of *Scn1b*^−/−^ neurons.

## Discussion

The outcome of GABAergic signaling is developmentally regulated in mammalian brain. Here, we investigated the timing of the developmental shift in GABAergic signaling in the *Scn1b*^−/−^ and *Scn1a*^+/−^ mouse models of DS. In both DS mouse models, maturation of GABAergic signaling in hippocampus and neocortex was delayed relative to WT. We propose that this delay contributes to epileptogenesis through principal neuron hyperexcitability. In addition to suggesting a novel mechanism of epileptogenesis in DS and potentially explaining altered antiepileptic efficacy in DS patients^31^, our results may impact the interpretation of previous *Scn1a* DS mouse model studies. A number of groups have demonstrated reduced firing of parvalbumin positive fast-spiking interneurons in *Scn1a* DS mouse brain at P14-20^9,10,32–34^. We show here that *E*_*GABA*_ is depolarized in *Scn1a*^+/−^ pyramidal neurons relative to WT from P13–21, suggesting that GABAergic currents are inward and excitatory. Based on our data, reduced interneuron firing at this time point is predicted to attenuate excitatory input to pyramidal neurons, resulting in hypoexcitability rather than disinhibition as previously proposed.

Excitatory GABAergic signaling is crucial for many developmental brain processes, including neurogenesis, cell migration, and network establishment. Depolarization of progenitor cells and immature neurons provides the main excitatory drive for cortical network formation during early brain development^21,35,36^. In addition, the depolarizing action of GABA plays important roles in controlling early network activity, including GDPs and promoting voltage-dependent, intrinsic neuronal bursting activity^37–39^. The subsequent maturation of GABAergic inhibition during the first two postnatal weeks in mice is required for the onset of the critical period of early postnatal brain development^40–43^, during which cortical circuits undergo extensive network rewiring and refinement. The delayed maturation of GABAergic signaling may delay the onset of the critical period and subsequently cortical network rewiring and maturation, which may contribute to the development of epileptogenesis and other aspects of DS such as intellectual disability or autism spectrum disorders.

How might *Scn1b* deletion or *Scn1a* haploinsufficiency lead to alterations in [Cl^−^]_i_? We found no differences in KCC2 (mRNA and protein) or NKCC1 (mRNA) expression in DS vs. WT brains, although we were not able to quantify NKCC1:KCC2 protein expression ratios between genotypes. We also found no evidence for β1 association with these CCCs in WT brain. VGSC β1 subunits are multifunctional^18^. In addition to modulation of channel gating, voltage-dependence, and cell surface expression, they contain an extracellular Ig domain that places them in the Ig superfamily of CAMs. Our body of work has demonstrated that β1 subunits function as CAMs to regulate critical processes in brain development including neuronal migration, cell-cell adhesion, and axonal fasciculation. As CAMs, β1 subunits associate with ECM and cytoskeletal scaffolding molecules^18^. Thus, even though we found no evidence for β1 association with KCC2 or NKCC1 in brain, it is possible that the absence of functional β1 subunits in neurons disrupts the homeostatic set point for [Cl^−^]_i_ through the dysregulation of other transporters or channels.

Treatment with the NKCC1 inhibitor bumetanide prolonged survival in a subset of *Scn1b*^−/−^ mice. Bumetanide administration in previous mouse studies and human clinical trials has led to conflicting results^44^. Bumetanide treatment led to suppression of epileptiform activity in rodent hippocampus^45^, reduction in seizure frequency and duration in human neonate^46^ and rodent seizure models^45,47^, and normalization of network activity in hippocampus and reduction of cortical excitability^27^. In contrast, a clinical trial examining the use of bumetanide to treat infantile spasms was unsuccessful^48^, and other studies have similarly failed to demonstrate seizure reduction in rodent models^49^. Here, while bumetanide delayed SUDEP in a subset of *Scn1b*^−/−^ mice, it did not prevent seizure activity and *Scn1b*^−/−^ mice still displayed severely low body weights. The mechanism underlying this effect is unclear. Bumetanide has poor penetration through the blood-brain barrier^28^. Studies show that less than 1% of systemically administered bumetanide reaches the brain, resulting in concentrations well below that required to block NKCC1^22^. Acute application of 10–20 μM bumetanide to brain slices in our study suppressed GABA-induced excitation, suggesting that poor bioavailability of bumetanide in the brain *in vivo* may explain why it did not prevent seizure activity. A recent review summarizes the diverse effects of bumetanide administration in animal models of acquired epilepsy^50^. However, to our knowledge, the present manuscript is the first demonstration of the effects of bumetanide in a model of epileptic encephalopathy with SUDEP. While the mechanisms of SUDEP remain unresolved, possible mechanisms include respiratory dysfunction, cardiac arrhythmia, autonomic imbalance, and postictal generalized electroencephlogram suppression with spreading depression to the brain stem^51^. Because *SLC12A2* is expressed in heart, peripheral nerve, and lung in addition to brain^52–55^, blocking NKCC1 function in these tissues may contribute to the observed SUDEP delay in our studies, although additional research is required. Nevertheless, our work suggests that therapeutic strategies targeting GABAergic signaling polarity in brain may be useful in reducing SUDEP risk in *SCN1A*- and *SCN1B*-DS.

## Methods

All methods were performed in accordance with the relevant guidelines and regulations of the University of Michigan.

### Animals

*Scn1b* WT and −/− mice were generated from *Scn1b*^+/−^ mice as described^8^. *Scn1b*^+/−^ mice were maintained on the C57BL/6 J background for over 20 backcrossed generations and are thus congenic. *Scn1a* WT and +*/−* mice, obtained from Dr. J. Kearney at Northwestern University, were generated as (129S6/SvEvTac × C57BL/6J)F1 hybrids as described^29^. Male and female animals were used in all experiments. Animals were housed in the Unit for Laboratory Animal Medicine at the University of Michigan. All procedures adhered to NIH guidelines and were approved by the University of Michigan Institutional Animal Care and Use Committee.

### Preparation of brain slices

Acute brain slices were prepared as described^13^. In brief, the brain was rapidly removed following euthanasia by isoflurane inhalation and decapitation. Coronal brain slices (200 µm) containing both neocortical and hippocampal components were prepared from P4–P19 *Scn1b* WT and −/− mice or P13–P21 *Scn1a* WT and +*/−* mice, as indicated in the figure legends, in ice-cold, oxygenated slicing solution saturated with 95% O_2_/5% CO_2_. The slicing solution contained (in mM): 110 sucrose; 62.5 NaCl; 2.5 KCl; 6 MgCl_2_; 1.25 KH_2_PO_4_; 26 NaHCO_3_; 0.5 CaCl_2_; and 20 D-glucose (pH 7.35–7.4 when saturated with 95% O_2_/5% CO_2_ at 22–25 °C). Slices were incubated initially in slicing solution for >40 min at RT followed by a 1:1 mixture of slicing solution and artificial cerebrospinal fluid (ACSF) in a holding chamber aerated continuously with 95% O_2_/5% CO_2_ at 25 °C for at least 30 min before use; ACSF contained (in mM): 125 NaCl; 2.5 KCl; 1 MgCl_2_; 1.25 KH_2_PO_4_; 26 NaHCO_3_; 2 CaCl_2;_ and 20 D-glucose (pH 7.35–7.4).

### Electrophysiological recording

The whole-cell patch clamp recording technique was used to examine spontaneous excitatory postsynaptic currents (sEPSCs) or GABA-evoked currents in pyramidal cells in neocortical and hippocampal slices. Each brain slice was transferred to a recording chamber where it was superfused (2–4 ml/min) with ACSF bubbled continuously with 95% O_2_/5% CO_2_. Pyramidal cells were visually identified based on their size, shape, and location using a NIKON E600FN upright microscope equipped with Nomarski optics (40x water immersion objective). For conventional whole-cell patch-clamp recording of sEPSCs, recording electrodes had a resistance of 3–6 MΩ when filled with a K-gluconate-based pipette solution that consisted of (in mM): 140 K-gluconate; 4 NaCl; 0.5 CaCl_2_; 10 HEPES; 5 EGTA; 2 Mg-ATP; and 0.4 GTP (pH 7.2–7.3, adjusted with KOH). sEPSCs were recorded from neurons in the presence or absence of 10 µM bicuculline (Sigma-Aldrich, St. Louis, MO) at a holding potential of −70 mV, as described^13^. With this low [Cl^−^]-containing internal solution and a holding potential of −70 mV, which is close to the predicted equilibrium potential for Cl^−^ (−83 mV), all inward currents were predicted to be glutamate receptor-mediated and bicuculline-insensitive in mature neurons. In the presence of bicuculline, neurons often generated huge inward sodium currents corresponding to spontaneous AP firing. sEPSCs shown in figures are selected from sections of recordings lacking AP firings. In a subset of experiments, APs were recorded in current-clamp recording mode before bicuculline treatment to measure the threshold for the initiation of APs. In experiments to examine GDPs in brain slices, K-gluconate in the pipette solution was replaced with equimolar N-methyl-D-glucamine (NMDG), as indicated in the figure legends.

For gramicidin perforated patch recordings of GABA-activated currents, the pipette solution contained (in mM): 145 KCl; 10 HEPES; 5 EGTA (pH 7.2–7.3, adjusted with KOH). A stock solution of 10 mg/ml of gramicidin D (Sigma-Aldrich) was prepared fresh in dimethylsulfoxide (DMSO). A fresh working solution of gramicidin (25–50 µg/ml) was prepared from the stock solution every two hours. Procedures for gramicidin perforated patch recording were similar to those described elsewhere^56^. In brief, the pipette tip was filled by dipping into gramicidin-free pipette solution for 30–60 s, and then backfilled with the same pipette solution containing 25–50 μg/ml gramicidin. After formation of a gigaohm seal, the progress of perforation was evaluated by monitoring changes in series resistance and resting membrane potential. GABA-mediated currents at holding potentials from −90 mV to −10 mV were evoked by a single 2 second pulse of GABA (100–200 µM) via a fine capillary glass probe that was positioned close to the target cell. GABA currents were recorded in the presence or absence of glutamate receptor antagonists 6-cyano-7-nitroquinoxaline-2,3-dione (CNQX, 10 µM) and amino-5-phosphonopentanoic acid (APV, 50–100 µM). A minimum 2 min interval between sequential GABA pulses was applied to allow full recovery of GABA_A_ receptors from desensitization. In most cases where cells fired too many action potentials at depolarized holding potentials, thereby interfering with the measurement of GABA-evoked currents, a full series of recordings was made with 0.5 µM tetrodotoxin (TTX) in the bath. GABA application was programmed and controlled by a VC-8 Perfusion Valve Controller system (Warner Instruments Corp., Hamden, CT). Signals were amplified with a Multiclamp 700B amplifier (Molecular Devices, Sunnyvale, CA), filtered at 2–4 kHz, and digitized at 20 kHz for offline analysis. Data were acquired with a Digidata 1440A interface and analyzed using pClamp10. The mean frequency and amplitudes of sEPSCs were analyzed using Mini Analysis 6.0.3 programs. The reversal potential for GABA-evoked currents (*E*_*GABA*_) was calculated according to *E*_*GABA*_ = −intercept (I_−20 mV_:I_−90 mV_,V_−20 mV_:V_−90 mV_)/slope(I_−20 mV_:I_−90 mV_,V_−20 mV_:V_−90 mV_), where I_−20 mV_:I_−90 mV_ is the GABA currents activated at a voltage range varying from −90 mV to −20 mV, i.e. V_−20 mV_:V_−90 mV_. In a subset of gramicidin perforated patch recordings, TTX was omitted in order to examine the effect of bumetanide *in vitro* on GABA-induced firing in brain slices. In those experiments, after stable baseline recordings of GABA-evoked firing, 10–20 µM bumetanide was added to the bath, prepared from a stock solution of 25 mM bumetanide dissolved in ethanol. The final concentration of ethanol was 0.4%, which has no significant effect on spontaneous and GABA responses. All experiments were performed at 22–25 °C.

### Preparation of Brain Membranes

Brain membrane proteins were prepared for use in Western blot analysis and co-immunoprecipitation as described^57^. Briefly, animals were anaesthetized with isoflurane, decapitated, brains were dissected with brainstem and cerebellum removed, then rapidly frozen in 2-methylbutane and stored at −80 °C until use. Frozen brains were placed into ice-cold Tris-EGTA (50 mM Tris, 10 mM EGTA, pH 8.0) containing Complete protease inhibitors at 2X the recommended concentration (Roche Diagnostics). From this point on, samples were kept on ice. Tissue was homogenized using a polytron homogenizer followed by 20 strokes using a glass Dounce homogenizer. Homogenates were centrifuged at 2500 × g for 10 min at 4 °C, and the supernatant was then centrifuged at 377,000 × g in a Beckman-Coulter MLA-130 rotor for 15 minutes at 4 °C. Membranes were resuspended in Tris-EGTA plus protease inhibitors, sonicated, and aliquots stored at −80 °C until use.

### Co-Immunoprecipitation

Protein A-sepharose 4B Fast Flow beads (Sigma-Aldrich) were washed three times in PBS and incubated overnight with end-over-end mixing at 4 °C with antibodies diluted in dilution buffer (60 mM Tris-HCl pH 7.5, 180 mM NaCl, 1% Triton X-100, 6 mM EDTA) to prepare antibody-conjugated beads. For anti-β1, anti-KCC2, and anti-NKCC1, 5 µl of antibody was used; for IgG control tubes, 1 µg of non-immune rabbit IgG (Jackson ImmunoResearch) was used. Brain membrane proteins were thawed and diluted in dilution buffer containing Complete protease inhibitors and 1X octyl-β-D-glucopyranoside (β-OG, Sigma-Aldrich) to solubilize detergent-resistant membranes. Tubes were centrifuged at 5000 × g for 5 min to remove insoluble material. Each tube contained ~1 mg of membrane proteins. Diluted membranes were then pre-cleared by end-over-end incubation with non-conjugated, washed protein-A sepharose beads for 1 h at 4 °C. Beads were pelleted by centrifugation at 3000 x g for 2 min, and the cleared supernatant was then incubated for 4–6 h at 4 °C with the antibody-conjugated beads in dilution buffer containing 1X β-OG. Beads were washed 3 times with ice-cold washing buffer (50 mM Tris-HCl pH 7.5, 150 mM NaCl, 5 mM EDTA, 0.02% SDS) containing protease inhibitors and 0.1% Triton X-100, then washed once with washing buffer without Triton X-100. Beads were pelleted and SDS sample buffer was added to each tube. Samples were heated at 37 °C for 30 min to elute proteins from beads and stored at −20 °C until use in Western blotting experiments.

### Western Blot Analysis

Western blot analysis of brain membrane proteins or co-immunoprecipitation samples was performed as described^58^. Briefly, protein concentrations were determined using a BCA Protein Assay kit (ThermoFisher Scientific). SDS sample buffer was added to membrane proteins, and either heated at 85 °C for 10 minutes (for β1 or NKCC1) or at 37 °C for 30 minutes (for KCC2). Samples were separated on hand-poured SDS-PAGE gels (7.5% for KCC2 and NKCC1, 10% for β1) and transferred to nitrocellulose then blocked for at least 2 h at RT. Blocking solution for anti-KCC2 blots contained 5% non-fat dry milk (NFDM) in TBS (0.1 M Tris-Cl, 0.5 M NaCl, pH 7.5). Blocking solution for anti-β1, anti-NKCC1, or anti-α-tubulin blots contained 5% NFDM and 1% bovine serum albumin in TBS + 0.1% Tween-20 (TBS-T). All primary and secondary antibodies were diluted in blocking solution except secondary antibodies for *Scn1a* Western blots which were diluted in TBS-T. Primary antibodies were incubated overnight at 4 °C, and secondary antibodies were incubated 1–2 h at RT. Wash steps of 4 × 15 min were performed after each antibody incubation step with either TBS-T (anti-β1, anti-NKCC1, or anti-α-tubulin) or TBS (anti-KCC2). Amersham ECL Prime or ECL Select Western Blotting Detection Reagents (GE Healthcare) or SuperSignal West Femto or West Dura (ThermoFisher Scientific) were used for detection. Chemiluminescent signal was acquired using a LiCor Odyssey Fc imaging system. Quantification of immunoreactive bands on Western blots was performed via densitometry using NIH ImageJ or LiCor ImageStation 5.2.5 software. Band density values (KCC2 or β1) were first normalized to the α-tubulin loading control for quantification. Relative protein expression was then normalized to the mean WT value on that blot and normalized values were combined across experiments. Sample sizes are reported in the text and all experiments were run twice as replicates.

### Immunohistochemistry

*Scn1b* WT and −/− mice (P10 or P17) were anesthetized using isofluorane then transcardially perfused with ~10 ml PBS followed by ~10 ml 4% paraformaldehyde (PFA) and brains dissected. Brains were post-fixed overnight in 4% PFA then cryoprotected in 10% sucrose followed by 30% sucrose overnight, flash frozen in 2-methylbutane, and stored at −80 °C. 20 μm sections were cut on a Leica cryostat and stored at −20 °C until processing for immunohistochemistry.

For immunohistochemistry, sections were dried and post-fixed for 10 min with 4% PFA, washed 3 times for 5 min each with 0.05 M phosphate buffer (PB), and incubated in blocking buffer (10% goat serum and 0.3% Triton X-100 in 0.1 M PB) for ≥2 h in a humidified chamber. Sections were then incubated with primary antibodies (diluted in blocking buffer) overnight in a humidified chamber and washed 3 times for 10 min with 0.1 M PB. From this point all steps were performed in the dark to minimize photobleaching of secondary antibodies. Sections were incubated with AlexaFluor-conjugated secondary antibodies (diluted in blocking buffer) for 2 h, washed 3 times for 10 min in 0.1 M PB, dried, and coverslips were mounted using ProLong Gold anti-fade reagent (Life Technologies).

Images were acquired using a Nikon A1R confocal microscope with Nikon NIS-Elements Advanced Research software located in the University of Michigan Department of Pharmacology using a 60x NA 1.40 oil objective. Confocal images spanning 2.5 μm (CA3) or 1.25 µm (cortical layers II/III) were acquired at 0.125 μm intervals and flattened using maximum signal in NIH ImageJ.

### Real-time Quantitative (q) RT-PCR

Mice (P17 *Scn1b* mice; P16 and P22 *Scn1a* mice) were anesthetized with isoflurane and brains were immediately dissected with cerebellum and brainstem removed. Total RNA was extracted from samples using TRIZOL reagent. Complementary DNA was created from 1 µg of total RNA using either the High Capacity cDNA Reverse Transcription Kit (Applied Biosystems) or the Superscript III First-Strand Synthesis System (ThermoFisher Scientific). Real-time qRT-PCR was performed using TaqMan Gene Expression Assays (ThermoFisher Scientific) for *Slc12a5* (KCC2, Mm00803929_m1), *Slc12a2* (NKCC1, Mm01265951_m1), *Scn1a* (Na_v_1.1, Mm00450580_m1), *Scn1b* (β1, Mm00441208_m1), *Gapdh* (GAPDH, Mm99999915_g1), and *Tbp* (Tata box binding protein, Mm00446971_m1). Experiments were run for 40 cycles using a 7900HT Real-Time PCR System (Applied Biosystems) located in the University of Michigan DNA Sequencing Core. Sequence Detection System 2.4 software (Applied Biosystems) was used for analysis. No detectable signals were observed in the no template controls. *Tbp* was used as the reference gene and relative transcript levels were determined using the 2^−ΔΔCT^ method; some sample sets were also analyzed using *Gapdh* as the reference gene. There were no differences between normalized results using *Tbp* versus *Gapdh*. Relative transcripts were normalized to mean WT values. Sample sizes for each experiment are reported in the text and all samples were run in triplicate.

### RNAseq

RNA was isolated from micro-dissected cortical layer VI tissue from P10 *Scn1b* WT and −/− mice using the Qiagen RNeasy Plus Kit according to manufacturer’s instructions. Cells were lysed through a sterile, 18-gauge hypodermic needle. The University of Michigan DNA Sequencing Core converted RNA to cDNA libraries using the TrueSeq Kit (Illumina) and sequenced using the Illumina HiSeq4000 platform with 50 cycles of paired end sequencing as fee for service^59^. Quality of reads were checked for each sample using FastQC (version v0.11.3). DeSEQ2 analysis was completed as fee for service through the University of Michigan Bioinformatics Core. Genes and transcripts were identified as differentially expressed based on the following criteria: test status = “OK”, false discovery rate $\le$ 0.5, and fold change ≥±1.5. UCSC mm10.fa was used as the reference genome sequence.

### Antibodies

Antibodies used for Western blotting and co-immunoprecipitation experiments were as follows, with dilutions used for Western blotting: rabbit anti-KCC2, Millipore, 1:1000; mouse anti-α-tubulin, Cedarlane Laboratories, 1:10,000; rabbit anti-β1 catalogue #13950, Cell Signaling Technologies, 1:1000; rabbit anti-NKCC1, Cell Signaling Technologies 1:1000; mouse anti-NKCC1 clone T4, Developmental Hybridoma Studies Bank, 1:500. For Western blotting experiments, secondary antibodies were HRP-conjugated goat anti-rabbit or goat anti-mouse as appropriate, ThermoFisher Scientific, 1:500. For immunofluorescent experiments, primary antibodies were rabbit anti-KCC2, Millipore, 1:500, and guinea pig anti-NeuN, Millipore, 1:1000; secondary antibodies were AlexaFluor 488 goat anti-rabbit and AlexaFluor 568 goat anti-guinea pig, Molecular Probes.

### Bumetanide treatment

Mice were injected subcutaneously twice daily (with a minimum of 8 h between injections) with 0.2 mg/kg bumetanide (West-Ward, Eatontown, NJ) diluted in sterile phosphate buffered saline (PBS) immediately before use. Injections were performed beginning at P0-P1 and continued through the lifespan of all *Scn1b*^−/−^ mice in each litter. Control animals were injected with sterile PBS at a volume equivalent to that used for bumetanide-treated animals of the same weight and monitored through the lifespan of all *Scn1b*^−/−^ mice in a given litter. Untreated control *Scn1b* litters did not receive any injections and were monitored for survival through the lifespan of all *Scn1b*^−/−^ mice in the litter.

### Clinical data

Patient data were obtained with informed consent from patients and parents following Institutional Review Board approval of the research protocol, KFSHRC RAC #2121053, at King Faisal Specialist Hospital and Research Center, Riyadh, Saudi Arabia as previously published^17^.

### Data analysis

Data are expressed as mean ± standard error of mean (SEM) unless otherwise indicated. Differences between *Scn1b* WT and −/− or *Scn1a* WT and +*/−* mice were analyzed statistically using Student’s *t* test, unpaired *t* test, Mann-Whitney test, or one-way ANOVA as appropriate. Dunnett’s procedure was used for *post hoc* comparison. Differences were considered significant at *p* < 0.05. Mouse survival was analyzed by Kaplan-Meier Log Rank (Mantel-Cox) test.

### Accession Titles

GSE128573: Differential gene expression in postnatal day 10 *Scn1b*^*+/+*^ vs. *Scn1b*^*−/−*^ cortical layer VI. GSM3680403: 2324 KO. GSM3680404: 2336 KO. GSM3680405: 2337 KO. GSM3680406: 2338 KO. GSM3680407: 2333 WT. GSM3680408: 2771 WT. GSM3680409: 2772 WT. GSM3680410: 2872 WT.

## Supplementary information

Supplementary Information

## Data Availability

The datasets generated during the current study are available in the NCBI Gene Expression Omnibus repository (https://www.ncbi.nlm.nih.gov/geo). See the Accession Titles above.
