# Supplementary material for: Delayed maturation of GABAergic signaling in the Scn1a and Scn1b mouse models of Dravet Syndrome
Source: Sci Rep. 2019 Apr 17;9:6210. doi: 10.1038/s41598-019-42191-0 (PMC6470170; doi:10.1038/s41598-019-42191-0)

Supplementary Information:

Delayed maturation of GABAergic signaling in the *Scn1a* and *Scn1b* mouse models of Dravet Syndrome

Yukun Yuan[1*], Heather A. O'Malley[1*], Melissa A. Smaldino[1#], Alexandra A. Bouza[1], Jacob M. Hull[2], and Lori L. Isom[1,2**]

[1]Department of Pharmacology and [2]Neuroscience Graduate Program, University of Michigan, Ann Arbor, MI 48109-5632

*These authors contributed equally to the work.

#Present address: Department of Biology, Ball State University, Muncie, IN 47306

**Corresponding Author: lisom@umich.edu, 734-936-3050

Supplementary Figures

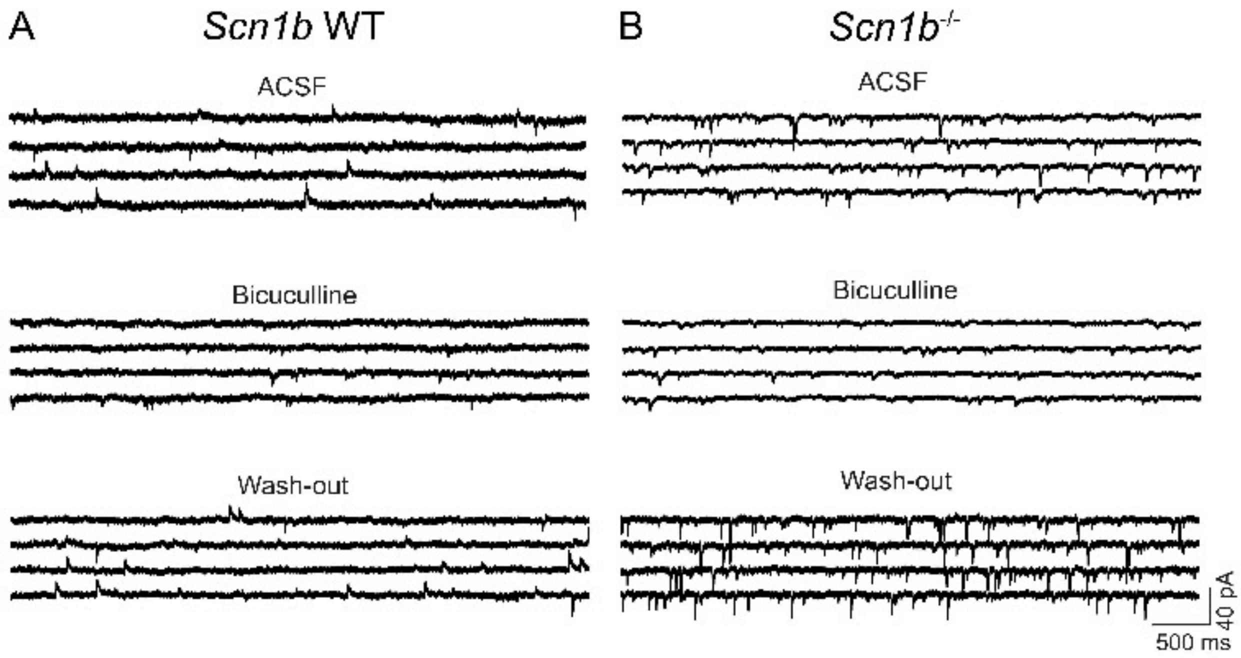

Fig. S1: P17 *Scn1b*$^{-/-}$ brain shows bicuculline-sensitive spontaneous inward currents. A: Representative spontaneous current events recorded with low $[Cl^-]_i$ in a CA1 pyramidal cell of a hippocampal slice from a P17 WT mouse in the absence (top) or presence (middle) of 10 µM bicuculline, and following washout with ACSF (bottom). The upward events were bicuculline-sensitive. Similar responses were also observed in cortical layer II/III neurons (data not shown). **B**: Representative spontaneous inward currents recorded with low $[Cl^-]_i$ in a layer II/III pyramidal cell of a cortical slice from a P16 *Scn1b*$^{-/-}$ mouse in the absence (top) and presence of 10 µM bicuculline (middle). Washout of bicuculline completely recovered spontaneous inward currents (bottom), demonstrating that the disappearance of these bicuculline-sensitive inward currents was not due to current rundown or other artifacts. Data are representative of 9 cells from 5 WT and 16 cells from 12 *Scn1b*$^{-/-}$ mice.

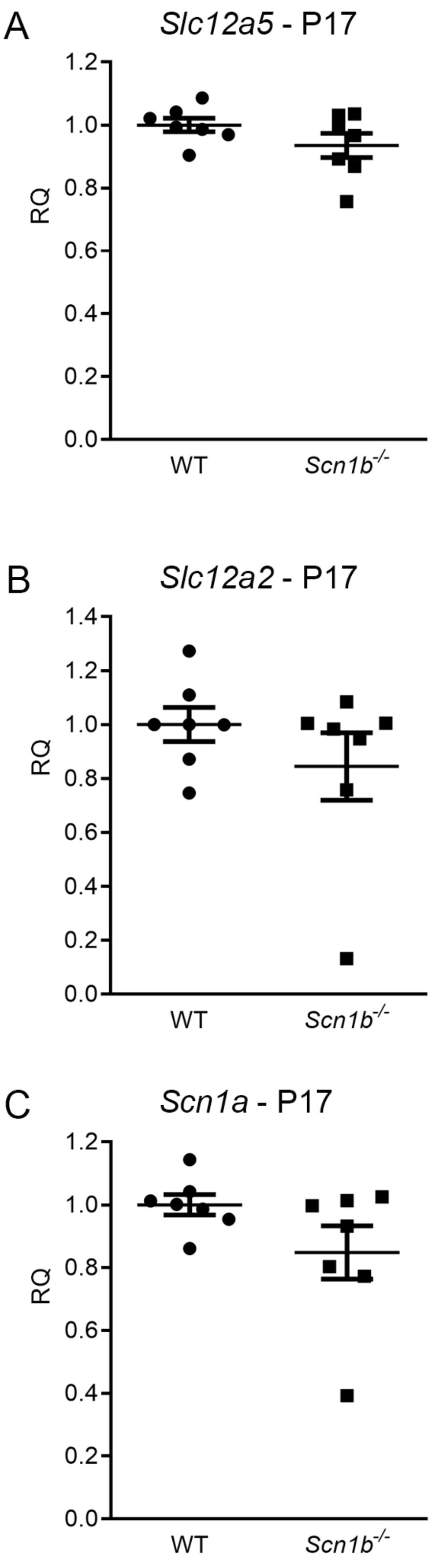

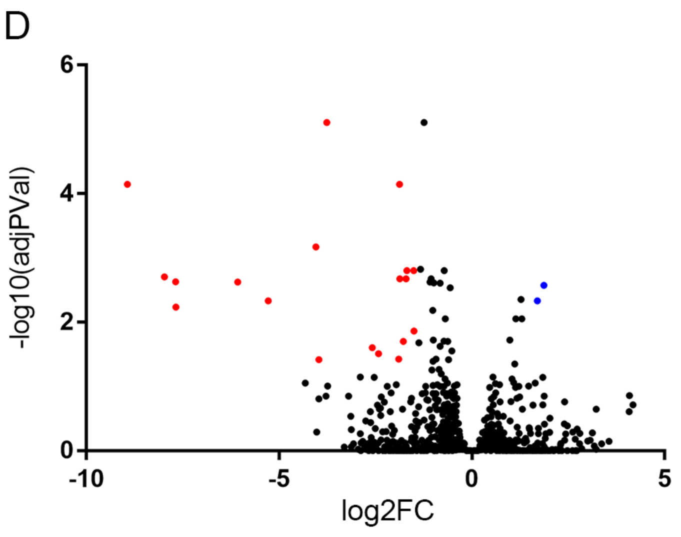

E

Gene	Log2FC	Adjusted P value
Eif2s3y	-8.938	7.16e-5
Ddx3y	-7.975	0.002
Uty	-7.689	0.002
Kdm5d	-7.682	0.006
Cpz	-6.074	0.002
Txndc3	-5.281	0.005
Pf4	-4.045	0.001
1700056E22Rik	-3.967	0.038
1500015O10Rik	-3.767	7.81e-6
Itga2	-2.587	0.025
Ebf2	-2.425	0.031
Pkd2l2	-1.898	0.038
Bmp6	-1.878	7.16e-5
Col3a1	-1.869	0.002
Pcolce	-1.780	0.020
Col1a2	-1.712	0.002
Spon2	-1.691	0.002
Igf2	-1.512	0.002
Thbd	-1.504	0.014
Mybpc3	1.864	0.003
A930011O12Rik	1.696	0.005
**NKCC1 (SLC12A2)**	**0.163**	**1**
**KCC2 (SLC12A5)**	**0.106**	**1**
**WNK1**	**0.170**	**1**
**STK39**	**-0.025**	**1**

Fig. S2: mRNA transcript levels and RNAseq results from *Scn1b* brain. **A-C**: Relative mRNA transcript levels of **A.** *Slc12a5* (KCC2), **B.** *Slc12a2* (NKCC1), or **C.** *Scn1a* ($Na_v1.1$) are not different between *Scn1b* WT and -/- brains at P17. Data are shown relative to WT. RQ: Relative quantity. **D**: Volcano plot showing differentially expressed genes in P10 *Scn1b* WT or -/- cortical layer VI. Red: Downregulated genes, Blue: upregulated genes (log2FC ⩾ 1.5 and false discovery rate ⩽ 0.05). **E:** Table showing genes differentially expressed between *Scn1b* WT and -/- cortical layer VI. In addition, there were no differences in expression between genotypes for the genes encoding NKCC1, KCC2, WNK1, or STK39.

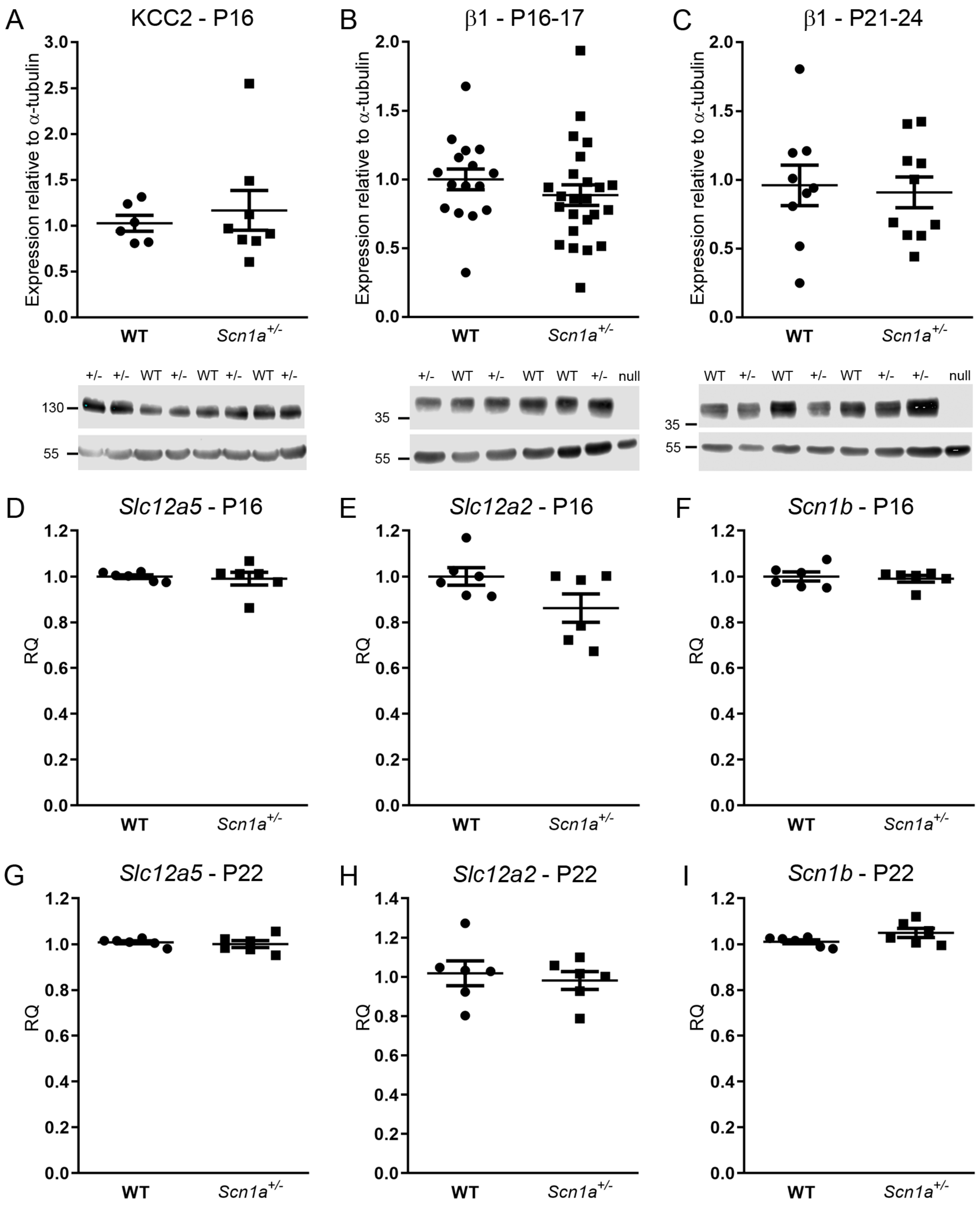

Fig. S3: Protein expression of KCC2 or β1 and mRNA expression of *Slc12a5*, *Slc12a2*, or *Scn1b* in *Scn1a* WT or +/- mouse brain. A-C: Protein expression levels of KCC2 and β1 are not different between *Scn1a* WT and +/- mouse brains. **A**: KCC2 protein expression at P16. Top: Quantification of KCC2 expression in *Scn1a* WT vs +/- brain,

normalized to α-tubulin expression and shown relative to WT. Bottom: Representative Western blot image. **B**: β1 protein expression at P16-17. Top: Quantification of β1 expression in *Scn1a* WT vs +/- brain, normalized to α-tubulin levels and shown relative to WT. Bottom: Representative Western blot image. **C**: β1 protein expression at P21-24. Top: Quantification of β1 expression in *Scn1a* WT vs +/- brain, normalized to α-tubulin levels and shown relative to WT. Bottom: Representative Western blot image. β1 null brain membranes were used as a negative control in **B** and **C**. **D-I**: Relative mRNA transcript levels of *Slc12a5* (KCC2), *Slc12a2* (NKCC1), and *Scn1b* (β1) are not different between *Scn1a* WT and +/- mice at P16 or P22. Data are shown relative to WT. **D**: Relative transcript levels of *Slc12a5* at P16. **E**: Relative transcript levels of *Slc12a2* at P16. **F**: Relative transcript levels of *Scn1b* at P16. **G**: Relative transcript levels of *Slc12a5* at P22. **H**: Relative transcript levels of *Slc12a2* at P22. **I**: Relative transcript levels of *Scn1b* at P22. RQ: Relative quantity.

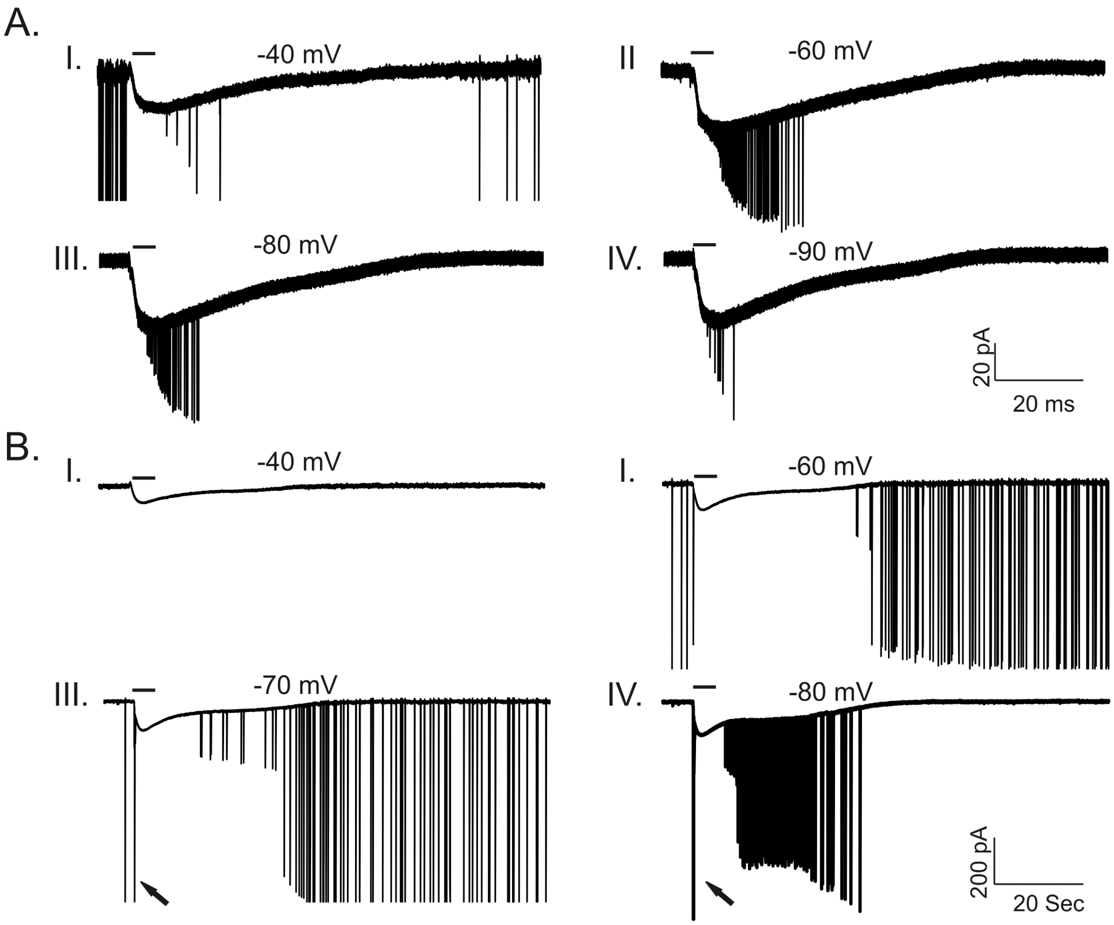

Fig. S4: **GABA induces excitatory responses in *Scn1a*$^{+/-}$ neurons. A**, **B**: Representative traces of GABA-evoked responses at different membrane holding potentials after establishing gramicidin perforated patch recording in two representative *Scn1a*$^{+/-}$ neurons. At membrane potentials more hyperpolarized than -60 mV (-70 and -80 mV are shown), puff application of GABA induced AP firing (arrows in **B-III** and **B-IV**). Each trace is representative of results from 6 individual mice.

Supplementary Tables:

Patient	Variant	Growth Parameters						
		Age	Weight (kg)	Percentile	Height (cm)	Percentile	Head (cm)	Percentile
1	c.449-2A>G	6 months	7.6	50-75	63	10-25	42.5	50
		7 months	7.6	50	67.7	50	43	50
		9 months	8.5	50	66	50	44.5	50
		30 months	10.7	<5	81	<5	47	<5
2	c.449-2A>G	3 months	5.4	50	57	10	40	50
		8 months	8	25-50	68	25	43	50-75
3	c.355T>G: p.Y119D	10 years	11.52	<1	90	<1	43.5	<1

Table S1: Growth parameters of *SCN1B*-linked DS patients at multiple developmental milestones. Head: head circumference.

Original full-length images of Western blots are provided below:

Fig5A - KCC2

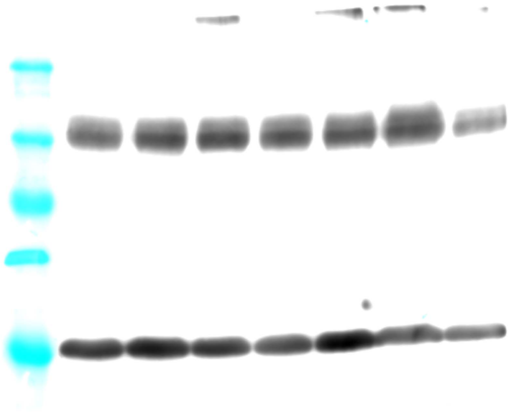

Fig. 5A - loading controls

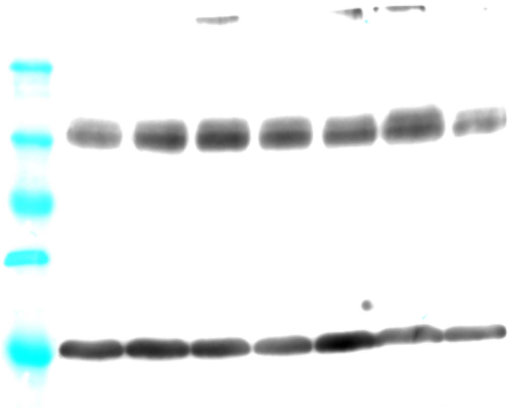

Fig. 5B, KCC2 and loading controls

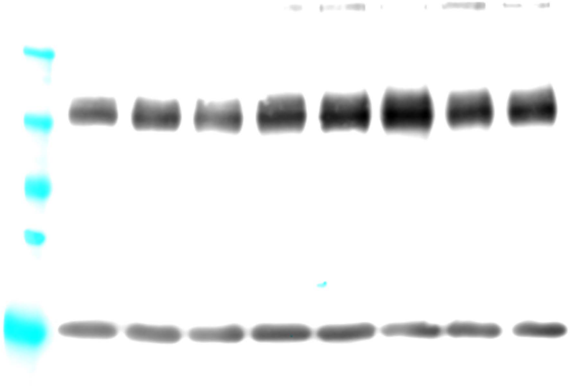

Fig. 5C - KCC2, lane 1 omitted

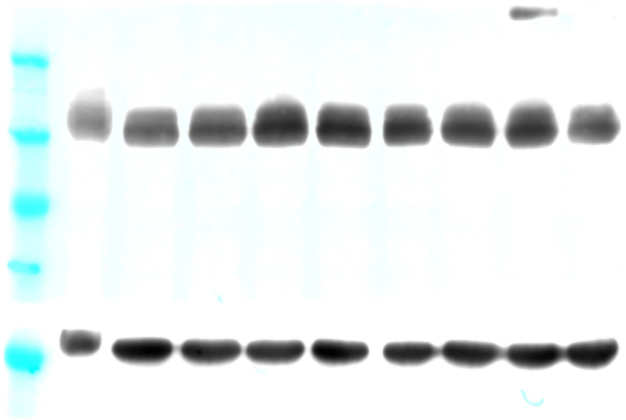

Fig. 5C - loading control, lane 1 omited

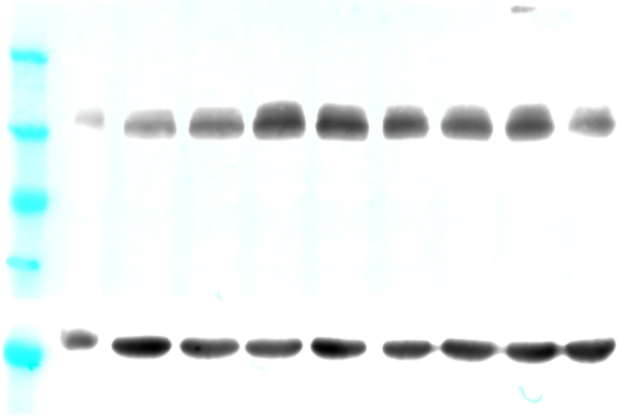

Fig. 5E, IP lanes and control

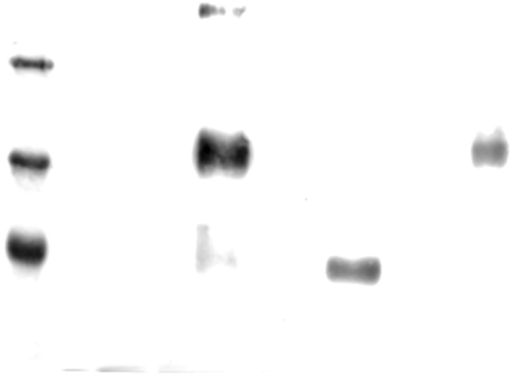

Fig. 5F - controls

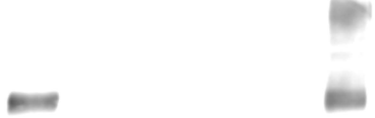

Fig. 5F - IP lanes

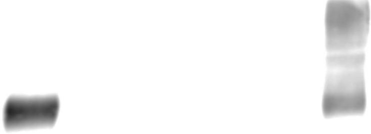

Fig. 5G - controls

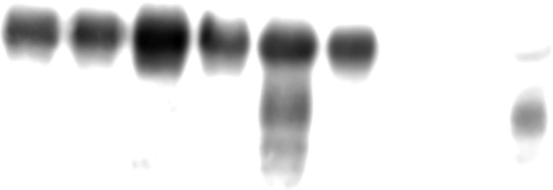

Fig. 5G - IP lanes

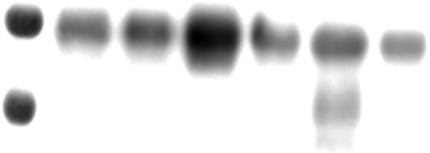

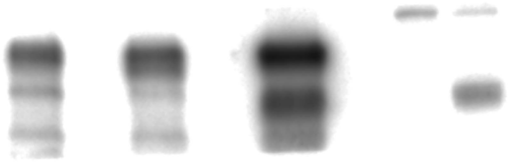

Fig. 5H - controls

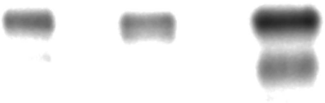

Fig. 5H - IP lanes

Supp. Fig. 4A - KCC2

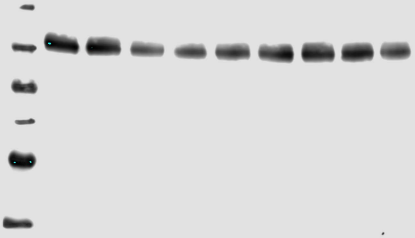

Supp. Fig. 4A - loading controls

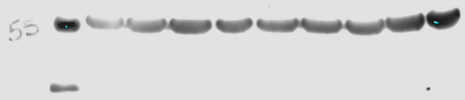

Supp. Fig. 4B - $\beta 1$, lanes 3-9 shown in figure

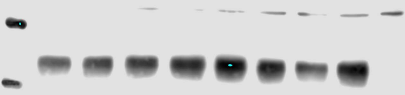

Supp. Fig. 4B - loading controls, lanes 3-9 shown

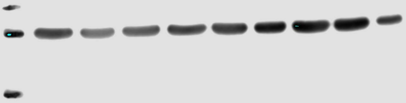

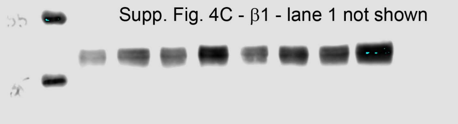
Supp. Fig. 4C - β1 - lane 1 not shown

Supp. Fig. 4C - loading controls, lane 1 not shown

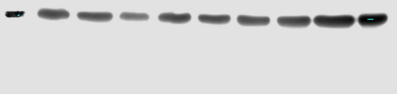

Supplement: Supplementary file 1 — Supplementary Information [file 41598_2019_42191_MOESM1_ESM.pdf]
